# Azole-Resistance in *Aspergillus terreus* and Related Species: An Emerging Problem or a Rare Phenomenon?

**DOI:** 10.3389/fmicb.2018.00516

**Published:** 2018-03-28

**Authors:** Tamara Zoran, Bettina Sartori, Laura Sappl, Maria Aigner, Ferran Sánchez-Reus, Antonio Rezusta, Anuradha Chowdhary, Saad J. Taj-Aldeen, Maiken C. Arendrup, Salvatore Oliveri, Dimitrios P. Kontoyiannis, Ana Alastruey-Izquierdo, Katrien Lagrou, Giuliana Lo Cascio, Jacques F. Meis, Walter Buzina, Claudio Farina, Miranda Drogari-Apiranthitou, Anna Grancini, Anna M. Tortorano, Birgit Willinger, Axel Hamprecht, Elizabeth Johnson, Lena Klingspor, Valentina Arsic-Arsenijevic, Oliver A. Cornely, Joseph Meletiadis, Wolfgang Prammer, Vivian Tullio, Jörg-Janne Vehreschild, Laura Trovato, Russell E. Lewis, Esther Segal, Peter-Michael Rath, Petr Hamal, Manuel Rodriguez-Iglesias, Emmanuel Roilides, Sevtap Arikan-Akdagli, Arunaloke Chakrabarti, Arnaldo L. Colombo, Mariana S. Fernández, M. Teresa Martin-Gomez, Hamid Badali, Georgios Petrikkos, Nikolai Klimko, Sebastian M. Heimann, Omrum Uzun, Maryam Roudbary, Sonia de la Fuente, Jos Houbraken, Brigitte Risslegger, Cornelia Lass-Flörl, Michaela Lackner

**Affiliations:** ^1^Division of Hygiene and Medical Microbiology, Medical University of Innsbruck, Innsbruck, Austria; ^2^Servei de Microbiologia, Hospital de la Santa Creu I Sant Pau, Barcelona, Spain; ^3^Microbiologia, Hospital Universitario Miguel Servet, IIS Aragon, Universidad de Zaragoza, Zaragoza, Spain; ^4^Department of Medical Mycology, Vallabhbhai Patel Chest Institute, University of Delhi, New Delhi, India; ^5^Microbiology Division, Department of Laboratory Medicine and Pathology, Hamad Medical Corporation, Doha, Qatar; ^6^Unit of Mycology, Department of Clinical Microbiology, Statens Serum Institute, Copenhagen University, Rigshospitalet, Copenhagen, Denmark; ^7^Department of Biomedical and Biotechnological Sciences, University of Catania, Catania, Italy; ^8^University of Texas MD Anderson Cancer Center, Houston, TX, United States; ^9^National Centre for Microbiology, Instituto de Salud Carlos III, Madrid, Spain; ^10^Department of Microbiology and Immunology, KU Leuven, Leuven, Belgium; ^11^Unità Operativa Complessa di Microbiologia e Virologia, Dipartimento di Patologia e Diagnostica, Azienda Ospedaliera Universitaria Integrata, Verona, Italy; ^12^Department of Medical Microbiology and Infectious Diseases, Canisius Wilhelmina Hospital, Nijmegen, Netherlands; ^13^Institute of Hygiene, Microbiology and Environmental Medicine, Medical University of Graz, Graz, Austria; ^14^Microbiology Institute, ASST Papa Giovanni XXIII, Bergamo, Italy; ^15^Infectious Diseases Research Laboratory, 4th Department of Internal Medicine, ATTIKON University Hospital, National and Kapodistrian University of Athens, Athens, Greece; ^16^Laboratorio Centrale di Analisi Chimico Cliniche e Microbiologia, IRCCS Foundation, Cà Granda Ospedale Maggiore Policlinico, Milan, Italy; ^17^Department of Biomedical Sciences for Health, Università degli Studi di Milano, Milan, Italy; ^18^Division of Clinical Microbiology, Department of Laboratory Medicine, Medical University of Vienna, Vienna, Austria; ^19^Institute for Medical Microbiology, Immunology and Hygiene, University of Cologne, Cologne, Germany; ^20^Mycology Reference Laboratory, Public Health England, Bristol, United Kingdom; ^21^Department of Laboratory Medicine, Karolinska Institutet, Karolinska University Hospital, Stockholm, Sweden; ^22^National Reference Medical Mycology Laboratory, Faculty of Medicine, Institute of Microbiology and Immunology, University of Belgrade, Belgrade, Serbia; ^23^Department I of Internal Medicine, Cologne Excellence Cluster on Cellular Stress Responses in Aging-Associated Diseases, Clinical Trials Centre Cologne, Center for Integrated Oncology (CIO Köln-Bonn), German Centre for Infection Research, University of Cologne, Cologne, Germany; ^24^Clinical Microbiology Laboratory, National Kapodistrian University of Athens, ATTIKON University Hospital Athens, Athens, Greece; ^25^Department of Hygiene and Medical Microbiology, Klinikum Wels-Grieskirchen, Wels, Austria; ^26^Department of Public Health and Pediatrics, Microbiology Division, Turin, Italy; ^27^Department I for Internal Medicine, University Hospital of Cologne, Cologne, Germany; ^28^German Centre for Infection Research, Partner Site Bonn-Cologne, Cologne, Germany; ^29^A.O.U. Policlinico Vittorio Emanuele Catania, Biometec–University of Catania, Catania, Italy; ^30^Infectious Diseases Unit, Department of Medical and Surgical Sciences, S. Orsola-Malpighi, University of Bologna, Bologna, Italy; ^31^Department of Clinical Microbiology and Immunology, Sackler School of Medicine, Tel Aviv University, Tel Aviv, Israel; ^32^Institute of Medical Microbiology, University Hospital Essen, University of Duisburg-Essen, Essen, Germany; ^33^Department of Microbiology, Faculty of Medicine and Dentistry, Palacky University Olomouc and University Hospital Olomouc, Olomouc, Czechia; ^34^Clinical Microbiology, Puerta del Mar University Hospital, University of Cádiz, Cádiz, Spain; ^35^Infectious Diseases Unit, 3rd Department of Pediatrics, Faculty of Medicine, Aristotle University School of Health Sciences, Hippokration General Hospital, Thessaloniki, Greece; ^36^Department of Medical Microbiology, Hacettepe University Medical School, Ankara, Turkey; ^37^Division of Mycology, Department of Medial Microbiology, Postgraduate Institute of Medical Education and Research, Chandigarh, India; ^38^Escola Paulista de Medicina, Federal University of São Paulo, São Paulo, Brazil; ^39^Departmento de Micología, Instituto de Medicina Regional, Universidad Nacional del Nordeste, CONICET, Resistencia, Argentina; ^40^Division of Clinical Mycology, Department of Microbiology, Vall d'Hebron University Hospital, Barcelona, Spain; ^41^Department of Medical Mycology and Parasitology, Invasive Fungi Research Center, Mazandaran University of Medical Sciences, Sari, Iran; ^42^School of Medicine, European University Cyprus, Nicosia, Cyprus; ^43^Department of Clinical Mycology, Allergy and Immunology, North Western State Medical University, Saint Petersburg, Russia; ^44^Department I for Internal Medicine, University Hospital of Cologne, Cologne, Germany; ^45^Department of Infectious Diseases and Clinical Microbiology, Hacettepe University Medical School, Ankara, Turkey; ^46^Department of Medical Mycology and Parasitology, School of Medicine, Iran University of Medical Science, Tehran, Iran; ^47^Department of Dermatology, Hospital Ernest Lluch Martin, Zaragoza, Spain; ^48^Department Applied and Industrial Mycology, Westerdijk Fungal Biodiversity Institute, Utrecht, Netherlands

**Keywords:** cryptic species, *Aspergillus* section *Terrei*, susceptibility profiles, azoles, *Cyp51A* alterations

## Abstract

**Objectives:** Invasive mold infections associated with *Aspergillus* species are a significant cause of mortality in immunocompromised patients. The most frequently occurring aetiological pathogens are members of the *Aspergillus* section *Fumigati* followed by members of the section *Terrei*. The frequency of *Aspergillus terreus* and related (cryptic) species in clinical specimens, as well as the percentage of azole-resistant strains remains to be studied.

**Methods:** A global set (*n* = 498) of *A. terreus* and phenotypically related isolates was molecularly identified (beta-tubulin), tested for antifungal susceptibility against posaconazole, voriconazole, and itraconazole, and resistant phenotypes were correlated with point mutations in the *cyp51A* gene.

**Results:** The majority of isolates was identified as *A. terreus* (86.8%), followed by *A. citrinoterreus* (8.4%), *A. hortai* (2.6%), *A. alabamensis* (1.6%), *A. neoafricanus* (0.2%), and *A. floccosus* (0.2%). One isolate failed to match a known *Aspergillus* sp., but was found most closely related to *A. alabamensis*. According to EUCAST clinical breakpoints azole resistance was detected in 5.4% of all tested isolates, 6.2% of *A. terreus sensu stricto (s.s.)* were posaconazole-resistant. Posaconazole resistance differed geographically and ranged from 0% in the Czech Republic, Greece, and Turkey to 13.7% in Germany. In contrast, azole resistance among cryptic species was rare 2 out of 66 isolates and was observed only in one *A. citrinoterreus* and one *A. alabamensis* isolate. The most affected amino acid position of the *Cyp51A* gene correlating with the posaconazole resistant phenotype was M217, which was found in the variation M217T and M217V.

**Conclusions:** *Aspergillus terreus* was most prevalent, followed by *A. citrinoterreus*. Posaconazole was the most potent drug against *A. terreus*, but 5.4% of *A. terreus sensu stricto* showed resistance against this azole. In Austria, Germany, and the United Kingdom posaconazole-resistance in all *A. terreus* isolates was higher than 10%, resistance against voriconazole was rare and absent for itraconazole.

## Introduction

In the last decade, the taxonomy and nomenclature of the previously morphologically defined genus *Aspergillus* changed, mainly due to comprehensive molecular phylogenetic studies and the introduction of the single name nomenclature (Samson et al., 2011, 2014; Alastruey-Izquierdo et al., 2013). With the introduction of molecular identification methods morphologically similar species were split into several cryptic species (Balajee et al., 2009a,b; Samson et al., 2011; Gautier et al., 2014). Samson et al. (2011) recognized 13 species in section *Terrei*: *A. terreus sensu stricto (s.s.), A. alabamensis, A. allahabadii, A. ambiguus, A. aureoterreus, A. carneus, A. floccosus, A. hortai, A. microcysticus, A. neoafricanus, A. neoindicus, A. niveus*, and *A. pseudoterreus*. In 2015, Guinea et al. (2015) described *A. citrinoterreus* as a new species of the section *Terrei* and subsequently *A. bicephalus* and *A. iranicus* were introduced (Arzanlou et al., 2016; Crous et al., 2016), resulting in a total of 16 accepted species.

*Aspergillus terreus s.s*., an important cause of fungal infections in immunocompromised patients, is reported as second or third most common pathogen of invasive aspergillosis (Baddley et al., 2003; Lass-Flörl et al., 2005; Blum et al., 2008). Treatment of infections caused by *A. terreus s.s*. and other section *Terrei* species (Walsh et al., 2003; Risslegger et al., 2017) may be difficult because of intrinsic amphotericin B resistance (Sutton et al., 1999; Escribano et al., 2012; Hachem et al., 2014; Risslegger et al., 2017). In addition, the emergence of *A. terreus sensu lato* (*s.l*.) isolates with reduced azole-susceptibility was reported (Arendrup et al., 2012; Won et al., 2017). Azole resistance in *A. terreus s.s*. and *A. fumigatus* is associated with mutations and alterations of the lanosterol-14-α steroldemethylase gene (*Cyp51A*), a key protein in the ergosterol biosynthesis pathway (Chowdhary et al., 2015, 2017). However, aside from mutations in the primary target gene, also other less known mechanisms (e.g., efflux pumps, overexpression of *cyp51*) were found to be involved in azole resistance (Arendrup, 2014; Rivero-Menendez et al., 2016).

The aim of this study was to evaluate the frequency of *A. terreus s.s*. and phenotypically similar (cryptic) species in a global set of clinical isolates and to screen for the presence of azole resistance.

## Materials and methods

### Fungal isolates

During an international *A. terreus* survey (Risslegger et al., 2017) various *A. terreus sensu lato (s.l.)* isolates were sent to and collected at the Medical University of Innsbruck by members of the ISHAM-ECMM-EFISG *TerrNet Study group* (www.isham.org/working-groups/aspergillus-terreus). Isolates were from Europe (*n* = 390), Middle East (*n* = 70), South America (*n* = 10), North America (*n* = 7), and South Asia (*n* = 19). A total of 498 strains, including isolates collected in Innsbruck within the last years, were analyzed (Supplementary Figure S1 and Supplementary Table S1), 495 were of clinical and 3 of environmental origin. For two isolates, the source is unknown. Isolates were cultured on Sabouraud's agar (Becton Dickinson, France), incubated at 37°C and stored in Sabouraud's broth with glycerin at −20°C.

### Antifungal susceptibility testing

Susceptibility to itraconazole, posaconazole, and voriconazole was determined by using reference broth microdilution according to EUCAST (www.EUCAST.org) and ETest® (bioMérieux, France). ETest® MICs were rounded to the next higher EUCAST concentrations and isolates displaying high MICs (≥0.25 mg/L for posaconazole, ≥2.0 mg/L for each, voriconazole and itraconazole) with ETest® were evaluated according to EUCAST. MIC_50_ and MIC_90_ were calculated for all studied section *Terrei* strains and each individual species. EUCAST clinical breakpoints (CBP) for *Aspergillus fumigatus* (see **Table 3**) were applied for wild typ and non-wildtyp categorization, as CBP for *Aspergillus terreus* are not available.

### Molecular identification

Genomic DNA was extracted by a method using CTAB (Lackner et al., 2012), and partial β-tubulin gene was amplified using bt2a/bt2b as previously described (Balajee et al., 2009a; Kathuria et al., 2015). KAPA2G Robust HotStart ReadyMix PCR Kit (Kapa Biosystems, USA) was used as master mix and PCR products were cleaned with ExoSAP-IT. For sequencing the BigDye XTerminator purification kit (Applied Biosystems, USA) was used. Sequencing was performed with the 3500 Genetic Analyzer (Applied Biosystems, USA) and data were analyzed with Bionumerics 6.6. Software (Applied Maths, Belgium). Generated sequences were compared with an in-house database of the Westerdijk Institute containing all available *Aspergillus* reference sequences.

### Sequencing of lanosterol 14-α sterol demethylase gene (*cyp51A*)

Azole-resistant isolates (**Table 3**) and a control set of susceptible isolates (Supplementary Table S2) underwent *Cyp51A* sequencing. *Cyp51A* genes were amplified by PCR, using KAPA2G Robust HotStart ReadyMix PCR Kit (Kapa Biosystems, USA) and in-house designed primers described by Arendrup et al. (2012). In short, PCR conditions were as follows: initial denaturation at 95°C for 5 min, followed by 35 cycles of 95°C for 1 min, 58°C for 1 min, 72°C for 2 min 30 s, and a final elongation step of 72°C for 10 min. Primers used for *Cyp51A* sequencing are provided in Supplementary Table S3. PCR products were cleaned with ExoSAP-IT and for sequencing the BigDye XTerminator purification kit was used. Sequencing was performed with the 3500 Genetic Analyzer and data were analyzed with Bionumerics 6.6. Software and Geneious 8 (Biomatters Limited).

## Results and discussion

### Epidemiology of cryptic species

Reports on cryptic species within the genus *Aspergillus* are on the rise (Balajee et al., 2009b; Alastruey-Izquierdo et al., 2013; Negri et al., 2014; Masih et al., 2016) and display variabilities in antifungal susceptibility (Risslegger et al., 2017). Negri et al. (2014) observed an increase of cryptic *Aspergillus* species causing fungal infections, and others calculated a prevalence of 10–15% of cryptic *Aspergillus* species in clinical samples (Balajee et al., 2009b; Alastruey-Izquierdo et al., 2013).

The present study analyzed a large number of isolates (*n* = 498) collected from Europe, Middle East, South America, North America, and South Asia (Supplementary Table S1 and Supplementary Figure S2) and identified *A. terreus* (*n* = 432), *A. citrinoterreus* (*n* = 42), *A. alabamensis* (*n* = 8), *A. hortai* (*n* = 13), *A. floccosus* (*n* = 1), and *A. neoafricanus* (*n* = 1). As previously reported (Risslegger et al., 2017) one isolate failed to be associated with any existing species, but clustered most closely to *A. alabamensis* (Supplementary Figure S1).

Our study showed limitations due to the unknown source and date of some clinical isolates. A differentiation between isolates from superficial and deep seeded infections was not made, therefore, source-variable resistance rates cannot be excluded. Number of studied isolates varied per country and might also introduce a bias to resistance rates.

*Aspergillus terreus s.s*. was the most prevalent species (86.8%), followed by *A. citrinoterreus* (8.4%), *A. hortai* (2.6%), and *A. alabamensis* (1.6%). This is in agreement with other authors (Balajee et al., 2009a; Neal et al., 2011; Escribano et al., 2012; Kathuria et al., 2015) showing that *A. terreus s.s*. is the most common species of section *Terrei* in clinical and environmental samples. In addition, we detected *A. floccosus* and *A. neoafricanus*. We did not identify *A. allahabadii, A. ambiguus, A. aureoterreus, A. bicephalus, A. carneus, A. iranicus, A. microcysticus, A. neoindicus, A. niveus*, and *A. pseudoterreus*. The reason for this might be that these species are less common in clinical samples and the environment. Our species distribution is in line with Kathuria et al. (2015), who reported for the first time a probable invasive aspergillosis and aspergilloma case due to *A. hortai*, which was found to occur in a prevalance of 1.4% of all section *Terrei* isolates. A multicenter study by Balajee et al. (2009a) observed a high frequency (33% of all clinical *A. terreus s.l*. isolates were *A. alabamensis*) of *A. alabamensis*. Other studies (Neal et al., 2011; Gautier et al., 2014; Risslegger et al., 2017) reported a lower prevalence of *A. alabamensis* isolates (up to 4.3%).

**Figure 1:**
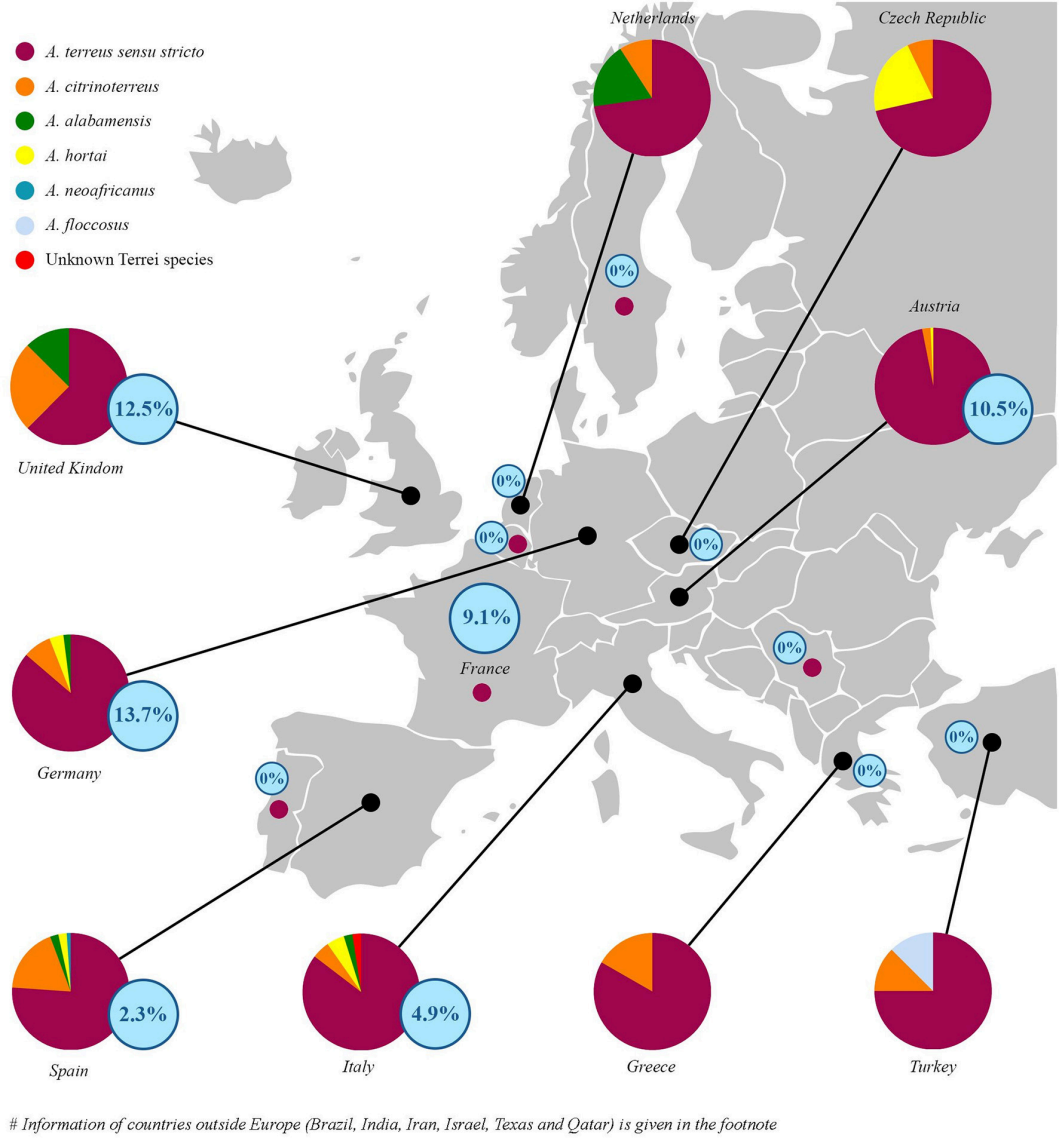
Epidemiological distribution of species (circles) and relative percentage of posaconazole resistance (according to EUCAST clinical breakpoints, see Table 2) isolates per country (blue numbers in blue circles) in respect to all investigated isolates. In France, Portugal, Serbia, and Sweden all collected isolates were identified as *A. terreus* sensu stricto (small dots in magenta). Azole-resistance percentage per countries are given in blue circled numbers. Species distribution in non-EU countries were as follows: India 100% *A. terreus s.s*.; Israel 84.85% *A. terreus s.s*. 12.12% *A. citrinoterreus* 3.03% *A. hortai*; Texas 80% *A. terreus s.s*. 10% *A. alabamensis* 10% *A. hortai*; Qatar: 83.34% *A. terreus s.s*. 16.66% *A. citrinoterreus*; Iran 63.64% *A. terreus s.s*. 36.36% *A. citrinoterreus*; and Brazil 85.71% *A. terreus s.s*., 14.29% *A. hortai*. All isolates from Iran, Israel, India, Brazil, Texas, and Qatar were susceptible to all azoles tested. For detailed information see Table 4.

Little is known about the geographical distribution of cryptic species of section *Terrei* in clinical specimens. *A. terreus s.s*. was exclusively found in France, Portugal, Serbia, India, and Sweden (Supplementary Table S1). Spain, Italy, Texas and Germany showed highest species diversity (Figure 1 and Supplementary Table S1). In Spain, the prevalent cryptic species were *A. citrinoterreus* (18.2%)*, A. alabamensis* (2.3%)*, A. hortai* (2.3%), and *A. neoafricanus* (1.1%), in Italy *A. citrinoterreus* and *A. hortai* (4.9%), together with one *A. alabamensis* (2.4%) and one unknown *Terrei* species (2.4%). In Germany *A. citrinoterreus* (7.8%) was followed by *A. hortai* (3.9%), and *A. alabamensis* (2.0%). In Texas 80.0% were *A. terreus s.s*. followed by 10% *A. alabamensis* and 10.0% *A. hortai*. Percentage of *A. citrinoterreus* was highest in Iran accounting 36.36% of all isolates (Figure 1).

### Azole resistance among studied section *Terrei* isolates

**Figure 2:**
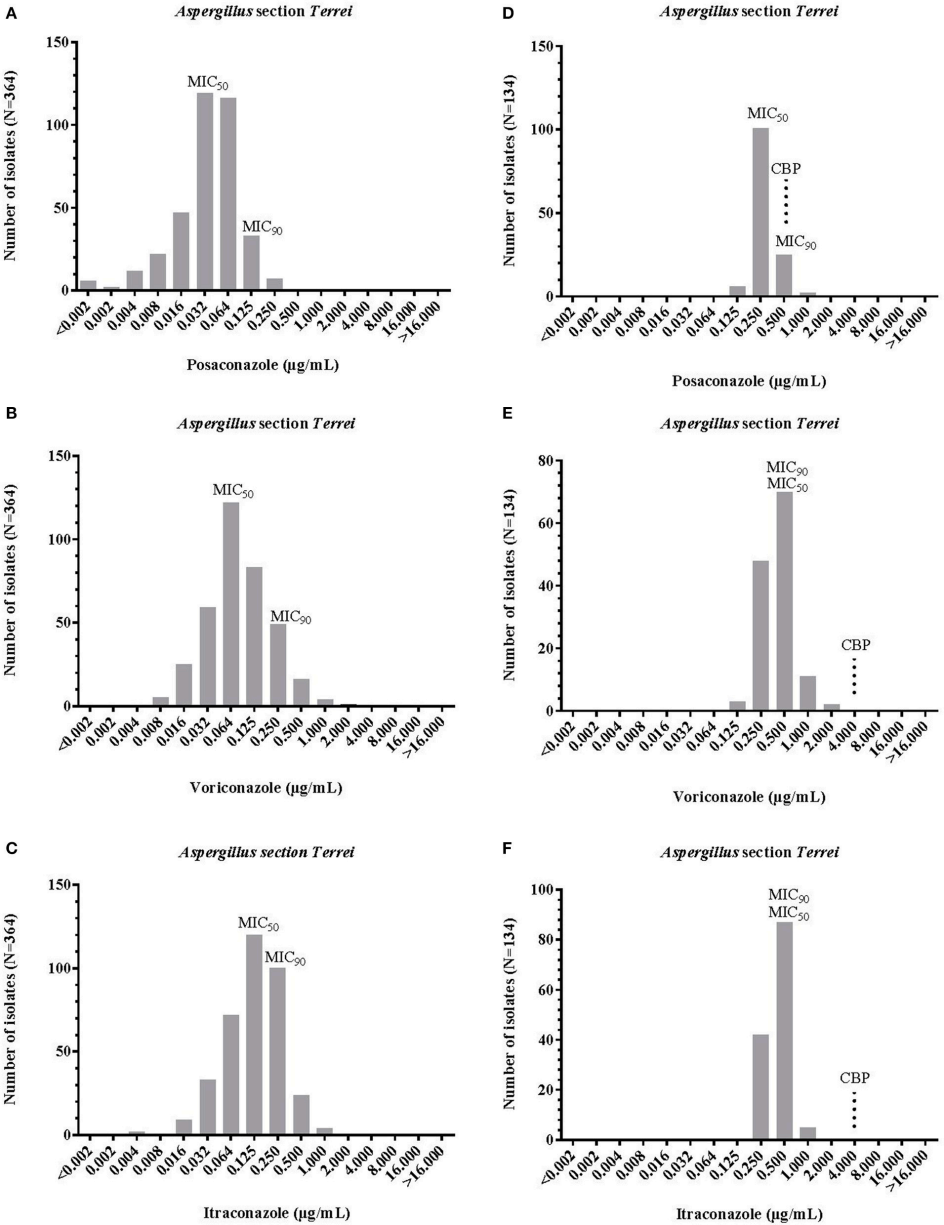
MIC distribution of posaconazole, itraconazole, voriconazole, and posaconazoleintraconazole against Aspergillus section Terrei, obtained by ETest® **(A-C)** and EUCAST method **(D-F)**. MIC, minimum inhibitory concentration; MIC_50_ and MIC_90_, MIC for 50 and 90% of tested population; CBP EUCAST clinical breakpoint (see Table 2).

Proposed epidemiological cut off values (ECOFF) values by EUCAST for *A. terreus s.s*. were 0.25 μg/mL for posaconazole, 2 μg/mL each for voriconazole and itraconazole. Antifungal susceptibility results (MICs) for *A. terreus s.s*. and cryptic species of the section *Terrei* are reported in Table 1 and Figure 2. Posaconazole had the lowest MICs for section *Terrei* isolates (MIC_50_, 0.032 μg/mL Etest® and 0.250 μg/mL EUCAST), followed by itraconazole (MIC_50_, 0.125 μg/mL Etest® and 0.500 μg/mL EUCAST), and voriconazole (MIC_50_, 0.064 μg/mL Etest® and 0.500 μg/mL EUCAST) (Figure 2). Lass-Flörl et al. (2009) observed similar MIC values for posaconazole among clinical isolates of *A. terreus s.l*. Astvad et al. (2017) tested *A. terreus* species complex isolates against voriconazole and observed slightly higher MIC ranges of 0.250–8.000 μg/mL.

**Table 1:** Clinical breakpoints according to EUCAST^1^.

**Antifungal agent**	**MIC S**	**(mg/L) R**
Posaconazole	≤0.125	>0.250
Voriconazole^*^	≤1.000	>2.000
Itraconazole	≤1.000	>2.000

1 http://www.eucast.org/clinical_breakpoints/

*MIC, minimum inhibitory concentration*;

* *CBPs are only available for Aspergillus fumigatus*.

No major differences in azole susceptibility profiles for *A. terreus s.s*. and cryptic species were observed (Table 2). Posaconazole and itraconazole MIC ranges for *A. terreus* were only slightly higher when compared to cryptic species. As shown in Table 2, MICs_50_ obtained with Etest® are equal among *A. terreus s.s*. isolates and cryptic species for posaconazole (0.032 μg/mL) and voriconazole (0.064 μg/mL). No significant differences in MIC_90_ values were observed among *A. terreus s.s*. isolates and cryptic species for itraconazole and posaconazole. Voriconazole MICs_90_ were somewhat higher among cryptic species (0.500 μg/mL) when compared to *A. terreus s.s*. (0.250 μg/mL). In general, all cryptic *A. terreus* species were per trend more susceptible to posaconazole and itraconazole than *A. terreus s.s*. The two most common cryptic species in our study, *A. citrinoterreus*, and *A. alabamensis*, showed highest MICs for voriconazole (range: 0.016–2.000 and 0.023–2.000 μg/mL).

**Table 2:** Antifungal susceptibility of *A. terreus s.s*. and related (cryptic) species (Balajee et al., 2009a,b; Samson et al., 2011; Gautier et al., 2014).

**Species**	**PSC (mg/L)**			**VRC (mg/L)**			**ITC (mg/L)**		
	**Range**	**MIC_50_**	**MIC_90_**	**Range**	**MIC_50_**	**MIC_90_**	**Range**	**MIC_50_**	**MIC_90_**
***A. terreus sensu stricto* (*n* = 432)**									
Etest® (*n* = 315)	<0.002–0.500	0.032	0.125	0.008–4.000	0.064	0.250	0.016–2.000	0.125	0.250
EUCAST (*n* = 117)	0.125–0.500	0.250	0.500	0.125–1.000	0.500	0.500	0.250–1.000	0.500	0.500
**Cryptic species (*****n*** = **66)**									
Etest® (*n* = 55)	<0.002–0.190	0.032	0.064	0.012–4.000	0.064	0.500	0.003–0.380	0.064	0.250
EUCAST (*n* = 11)	0.125–0.250	NA	NA	0.125–2.000	NA	NA	0.125–0.250	NA	NA

*Minimum inhibitory concentrations (MICs) of posaconazole, voriconazole, and itraconazole were obtained by ETest® and EUCAST method*.

*MIC, minimum inhibitory concentration; MIC_50_ and MIC_90_, MIC for 50 and 90% of tested population; ITC, itraconazole; VRC, voriconazole; POS, posaconazole; EUCAST, European Committee for Antimicrobial Susceptibility Testing; NA, not applicable; N, number of tested isolates*.

According to EUCAST breakpoints 5.4% of all section *Terrei* isolates are posaconazole resistant. This is a relatively high frequency in comparison to *A. fumigatus*. A prospective multicenter international surveillance study (van der Linden et al., 2015) showed a prevalence of azole-resistance of 3.2% in *A. fumigatus*. As shown in Table 3, only mono-azole resistance was observed (posaconazole, MICs ranged from 0.500 to 1.000 μg/mL). Azole resistance was more frequently observed among *A. terreus s.s*. isolates and was rare among cryptic species. One *A. citrinoterreus* isolate was resistant against posaconazole (0.500 μg/mL). Posaconazole resistant strains were detected from Germany (13.7%) followed by the United Kingdom (12.5%), Austria (10.5%), France (9.1%), Italy (4.9%), and Spain (2.3%) (Tables 3, 4 and Figure 1). In Turkey, Greece, Serbia, Iran, Israel, India, Brazil, Texas, and Qatar all isolates were susceptible against all azoles tested. However, resistance rates per countries might be influenced by multiple factors such as specimen handling and sampling, and investigated patient cohorts.

**Table 3:** Summary of mutations detected in azole-resistant *A. terreus and A. citrinoterreus*.

**Species**	**Isolate**	**EUCAST MIC(mg/L)**			**Mutation (NA)**	**Substitution (AA)**
		**VRC**	**ITC**	**POS**		
***A. terreus sensu stricto***						
(*n* = 26)	51	0.500	2.000	0.500	M217T	T650C
	10	0.500	0.250	0.500	No mutation	
	138	1.000	0.500	1.000	M217V, D344N	A649G, G1030A
	368	1.000	0.500	1.000	No mutation	
	T104	0.500	1.000	0.500	No mutation	
	T112	0.500	0.500	0.500	E319G	A956G
	T13	0.500	0.500	0.500	No mutation	
	T136	0.500	0.500	0.500	No mutation	
	T15	0.500	1.000	0.250	No mutation	
	T152	0.500	0.500	0.500	No mutation	
	T153	0.500	0.500	0.500	A221V	C662T
	T156	0.500	0.500	0.500	No mutation	
	T157	0.500	0.500	0.500	No mutation	
	T159	0.500	0.500	0.500	No mutation	
	T160	0.500	0.500	0.500	No mutation	
	T55	0.500	0.500	0.500	No mutation	
	T59	0.500	0.250	0.500	No mutation	
	T61	0.500	0.500	0.500	No mutation	
	T65	0.500	0.500	0.500	No mutation	
	T67	0.500	0.500	0.500	No mutation	
	T68	0.500	0.500	0.500	No mutation	
	T80	0.500	0.500	0.500	No mutation	
	T9	0.500	0.500	0.250	No mutation	
	T91	0.500	0.500	0.500	No mutation	
	T98	0.500	0.500	0.500	No mutation	
	16	0.500	1.000	1.000	No mutation	
***A. citrinoterreus***						
(*n* = 1)	150	0.500	0.500	1.000	I23T, R163H, E202D, Q270R	T69C, G489A, G607C, A810G

*Susceptibily was determined by EUCAST and resistance categorization was based on EUCAST clinical breakpoints (see Table 1)*.

*MIC, minimum inhibitory concentration; NA, nucleic acid; AA, Amino acid; ITC, itraconazole; VRC, voriconazole; POS, posaconazole: resistant strains based on the EUCAST Antifungal Clinical Breakpoints. EUCAST. European Committee for Antimicrobial Susceptibility Testing*.

**Table 4:** Posaconazole resistance per country relative to (1) all studied isolates and (2) *A. terreus s.s*. only (also see Figure 1).

**Country**	**All isolates studied (%)**	***A. terreus sensu stricto* (%)**
Austria	10.5	10.9
France	9.1	9.1
Germany	13.7	15.9
Italy	4.9	5.7
Spain	2.3	1.5
UK	12.5	12.5
Iran	0.0	0.0
Israel	0.0	0.0
India	0.0	0.0
Brazil	0.0	0.0
Texas	0.0	0.0
Qatar	0.0	0.0

Posaconazole showed to be the most effective azole against *A. terreus s.s*. and related (cryptic) species. However, a high frequency of posaconazole resistant isolates was detected and it was shown that the occurrence of azole resistance differed geographically. Posaconazole resistance among cryptic species was rare when compared to *A. terreus s.s*.

### SNPs in the *Cyp51A* gene

Mutations at the position M217 were reported to be associated with reduced susceptibility against itraconazole (MICs of 1.0–2.0 μg/mL), voriconazole (MICs of 1.0–4.0 μg/mL), and posaconazole (MICs of 0.25–0.5 μg/mL) (Arendrup et al., 2012), however the substituting amino acids varied from the one found in our study. Our isolates carried the mutations M217T (nucleic acid change T650C) or M217V (nucleic acid change A649G) (Table 3) and were exclusively resistant against posaconazole, when applying the EUCAST clinical breakpoints. Strains carrying the point mutation M217I in the study from Arendrup et al. (2012) were isolated from cystic fibrosis patients receiving long-term azole therapy and showed a pan-azole resistant phenotype. Another posaconazole resistant isolate (T153) carried an amino acid substitution at position A221V, a mutation, which was also previously reported by Arendrup et al. (2010), but was not associated with posaconazole resistance. Hence, functional studies in mutant strains are needed to evaluate the role of the mutations M217V, M217I, M217T, and A221V, which are all located in close proximity to the hot spot mutation M220I of *A. fumigatus*. Understanding the impact of mutations at the position M217 on the protein folding pattern and subsequently on binding capacities of azoles is the key to evaluate its role as azole-resistance markers. Other hotspot mutations, which were linked to acquired azole-resistance in *A. fumigatus*, are G54, L98, and M220 (Arendrup et al., 2010). None of them were found in our resistant isolates, suggesting different mechanisms of acquired azole-resistance than in *A. fumigatus*. The role of the other coding mutations within *A. terreus s.s*. isolates E19G (nucleic acid substitution A956G) and D344N (nucleic acid substitution C662T) remains to be studied. Voriconazole resistant *A. citrinoterreus* carried the amino acid changes I23T, R163H, E202D, Q270R (Table 3), which need to be analyzed in detail.

## Conclusions

*Aspergillus terreus s.s*. was most prevalent, followed by *A. citrinoterreus*. Posaconazole was the most potent azole against the investigated isolates and species. Approximately 5% of all tested *A. terreus s.s*. isolates were resistant against posaconazole *in vitro*. In Austria, Germany and the UK posaconazole resistance was higher than 10% in all *A. terreus s.s*. isolates. Resistance against itraconazole and voriconazole was rare.

## Author contributions

TZ: manuscript writing, Etest susceptibility testing, data analysis and interpretation, discussion of results, DNA extraction, sequencing; BS: wrote parts of the manuscript (M&M), DNA extraction, sequencing, nucleic acid alignments, and amino acid alignments; LS: EUCAST susceptibility testing, DNA extraction; JH: BLAST comparison of sequences, molecular species identification; BR: culturing of isolates, subcultivation of isolates, morphological identification, data management; CL-F: manuscript writing, discussion of results, clinical background, funding, coordination of the TerrNet study group, isolate recruitment; ML: manuscript writing, data analysis, study design, supervising TZ, BS, and LS; MA, FS-R, AR, AnC, ST-A, MA, SO, DK, AA-I, KL, GL, JM, WB, CF, MD-A, AG, AT, BW, AH, EJ, LK, VA-A, OC, JM, WP, VT, J-JV, LT, RL, ES, P-MR, PH, MR-I, ER, SA-A, ArC, ALC, MF, MM-G, HB, GP, NK, SH, OU, MR, SdlF are members of the EFISG-ISHAM-ECMM TerrNet Study group: providing strains and data.

### Conflict of interest statement

The authors declare that the research was conducted in the absence of any commercial or financial relationships that could be construed as a potential conflict of interest. The handling Editor declared a past co-authorship with several of the authors BS, KL, JM, BW, VA-A, OC, J-JV, P-MR, CL-F, and ML. The handling Editor declared a shared affiliation, and co-authorship, with one of the authors WB.
