# Corrigendum: Azole-Resistance in *Aspergillus terreus* and Related Species: An Emerging Problem or a Rare Phenomenon?

**DOI:** 10.3389/fmicb.2018.03245

**Published:** 2019-01-14

**Authors:** Tamara Zoran, Bettina Sartori, Laura Sappl, Maria Aigner, Ferran Sánchez-Reus, Antonio Rezusta, Anuradha Chowdhary, Saad J. Taj-Aldeen, Maiken C. Arendrup, Salvatore Oliveri, Dimitrios P. Kontoyiannis, Ana Alastruey-Izquierdo, Katrien Lagrou, Giuliana Lo Cascio, Jacques F. Meis, Walter Buzina, Claudio Farina, Miranda Drogari-Apiranthitou, Anna Grancini, Anna M. Tortorano, Birgit Willinger, Axel Hamprecht, Elizabeth Johnson, Lena Klingspor, Valentina Arsic-Arsenijevic, Oliver A. Cornely, Joseph Meletiadis, Wolfgang Prammer, Vivian Tullio, Jörg-Janne Vehreschild, Laura Trovato, Russell E. Lewis, Esther Segal, Peter-Michael Rath, Petr Hamal, Manuel Rodriguez-Iglesias, Emmanuel Roilides, Sevtap Arikan-Akdagli, Arunaloke Chakrabarti, Arnaldo L. Colombo, Mariana S. Fernández, M. Teresa Martin-Gomez, Hamid Badali, Georgios Petrikkos, Nikolai Klimko, Sebastian M. Heimann, Omrum Uzun, Maryam Roudbary, Sonia de la Fuente, Jos Houbraken, Brigitte Risslegger, Raquel Sabino, Cornelia Lass-Flörl, Michaela Lackner

**Affiliations:** ^1^Division of Hygiene and Medical Microbiology, Medical University of Innsbruck, Innsbruck, Austria; ^2^Servei de Microbiologia, Hospital de la Santa Creu I Sant Pau, Barcelona, Spain; ^3^Microbiologia, Hospital Universitario Miguel Servet, IIS Aragon, Universidad de Zaragoza, Zaragoza, Spain; ^4^Department of Medical Mycology, Vallabhbhai Patel Chest Institute, University of Delhi, New Delhi, India; ^5^Microbiology Division, Department of Laboratory Medicine and Pathology, Hamad Medical Corporation, Doha, Qatar; ^6^Unit of Mycology, Department of Clinical Microbiology, Statens Serum Institute, Copenhagen University, Rigshospitalet, Copenhagen, Denmark; ^7^Department of Biomedical and Biotechnological Sciences, University of Catania, Catania, Italy; ^8^University of Texas MD Anderson Cancer Center, Houston, TX, United States; ^9^National Centre for Microbiology, Instituto de Salud Carlos III, Madrid, Spain; ^10^Department of Microbiology and Immunology, KU Leuven, Leuven, Belgium; ^11^Unità Operativa Complessa di Microbiologia e virologia, Dipartimento di Patologia e diagnostica, Azienda Ospedaliera Universitaria Integrata, Verona, Italy; ^12^Department of Medical Microbiology and Infectious Diseases, Canisius Wilhelmina Hospital, Nijmegen, Netherlands; ^13^Institute of Hygiene, Microbiology and Environmental Medicine, Medical University of Graz, Graz, Austria; ^14^Microbiology Institute, ASST Papa Giovanni XXIII, Bergamo, Italy; ^15^Infectious Diseases Research Laboratory, 4th Department of Internal Medicine, ATTIKON University Hospital, National and Kapodistrian University of Athens, Athens, Greece; ^16^Laboratorio Centrale di Analisi Chimico Cliniche e Microbiologia, IRCCS Foundation, Cà Granda Ospedale Maggiore Policlinico, Milan, Italy; ^17^Department of Biomedical Sciences for Health, Università degli Studi di Milano, Milan, Italy; ^18^Division of Clinical Microbiology, Department of Laboratory Medicine, Medical University of Vienna, Vienna, Austria; ^19^Institute for Medical Microbiology, Immunology and Hygiene, University of Cologne, Cologne, Germany; ^20^Mycology Reference Laboratory, Public Health England, Bristol, United Kingdom; ^21^Department of Laboratory Medicine, Karolinska Institutet, Karolinska University Hospital, Stockholm, Sweden; ^22^National Reference Medical Mycology Laboratory, Faculty of Medicine, Institute of Microbiology and Immunology, University of Belgrade, Belgrade, Serbia; ^23^Department I of Internal Medicine, Cologne Excellence Cluster on Cellular Stress Responses in Aging-Associated Diseases, Clinical Trials Centre Cologne, Center for Integrated Oncology (CIO Köln-Bonn), German Centre for Infection Research, University of Cologne, Cologne, Germany; ^24^Clinical Microbiology Laboratory, National Kapodistrian University of Athens, ATTIKON University Hospital Athens, Athens, Greece; ^25^Department of Hygiene and Medical Microbiology, Klinikum Wels-Grieskirchen, Wels, Austria; ^26^Department of Public Health and Pediatrics, Microbiology Division, Turin, Italy; ^27^Department I for Internal Medicine, University Hospital of Cologne, Cologne, Germany; ^28^German Centre for Infection Research, Partner Site Bonn-Cologne, Cologne, Germany; ^29^A.O.U. Policlinico Vittorio Emanuele Catania, Biometec – University of Catania, Catania, Italy; ^30^Infectious Diseases Unit, Department of Medical and Surgical Sciences, S. Orsola-Malpighi, University of Bologna, Bologna, Italy; ^31^Department of Clinical Microbiology and Immunology, Sackler School of Medicine, Tel Aviv University, Tel Aviv, Israel; ^32^Institute of Medical Microbiology, University Hospital Essen, University of Duisburg- Essen, Essen, Germany; ^33^Department of Microbiology, Faculty of Medicine and Dentistry, Palacky University Olomouc and University Hospital Olomouc, Olomouc, Czechia; ^34^Clinical Microbiology, Puerta del Mar University Hospital, University of Cádiz, Cádiz, Spain; ^35^Infectious Diseases Unit, 3rd Department of Pediatrics, Faculty of Medicine, Aristotle University School of Health Sciences, Hippokration General Hospital, Thessaloniki, Greece; ^36^Department of Medical Microbiology, Hacettepe University Medical School, Ankara, Turkey; ^37^Division of Mycology, Department of Medial Microbiology, Postgraduate Institute of Medical Education and Research, Chandigarh, India; ^38^Escola Paulista de Medicina, Federal University of São Paulo, São Paulo, Brazil; ^39^Departmento de Micología, Instituto de Medicina Regional, Universidad Nacional del Nordeste, CONICET, Resistencia, Argentina; ^40^Division of Clinical Mycology, Department of Microbiology, Vall d'Hebron University Hospital, Barcelona, Spain; ^41^Department of Medical Mycology and Parasitology, Invasive Fungi Research Center, Mazandaran University of Medical Sciences, Sari, Iran; ^42^School of Medicine, European University Cyprus, Nicosia, Cyprus; ^43^Department of Clinical Mycology, Allergy and Immunology, North Western State Medical University, Saint Petersburg, Russia; ^44^Department of Infectious Diseases and Clinical Microbiology, Hacettepe University Medical School, Ankara, Turkey; ^45^Department of Medical Mycology and Parasitology, School of Medicine, Iran University of Medical Science, Tehran, Iran; ^46^Department of Dermatology, Hospital Ernest Lluch Martin, Zaragoza, Spain; ^47^Department Applied and Industrial Mycology, Westerdijk Fungal Biodiversity Institute, Utrecht, Netherlands; ^48^Reference Unit for Parasitic and Fungal Infections, Department of Infectious Diseases, National Institute of Health Coutor Ricardo Jorge, Lisbon, Portugal

**Keywords:** cryptic species, *Aspergillus* section *Terrei*, susceptibility profiles, azoles, *Cyp51A* alterations

Raquel Sabino was not included as an author in the published article. The authors apologize for this error and state that this does not change the scientific conclusions of the article in any way. The original article has been updated.

## Conflict of Interest Statement

The authors declare that the research was conducted in the absence of any commercial or financial relationships that could be construed as a potential conflict of interest.

