# Supplementary material for: Azole-Resistance in Aspergillus terreus and Related Species: An Emerging Problem or a Rare Phenomenon?
Source: Front Microbiol. 2018 Mar 28;9:516. doi: 10.3389/fmicb.2018.00516 (PMC5882871; doi:10.3389/fmicb.2018.00516)
Supplement: Supplementary file 1 [file DataSheet1.pdf]

Supplementary Material

Azole-resistance in *Aspergillus* section *Terrei* an emerging problem or a rare phenomenon?

Tamara Zoran et al.

Supplementary Table S1. Investigated isolates in study

Strain	Species	Origin	Specimen	First-line Therapy	Susceptibility testing
**1**	*A. terreus sensu stricto*	Italy	BAL	No data	ETest®
**2**	*A. terreus sensu stricto*	Italy	BAL	No data	ETest®
**3**	*A. citrinoterreus*	Italy	Toenail	No	ETest®
**4**	*A. terreus sensu stricto*	Italy	Toenail	No	ETest®
**5**	*A. terreus sensu stricto*	Italy	Toenail	No	ETest®
**6**	*A. terreus sensu stricto*	Italy	Toenail	No	ETest®
**7**	*A. terreus sensu stricto*	Italy	Toenail	No	ETest®
**8**	*A. terreus sensu stricto*	Italy	Toenail	No	ETest®
**9**	*A. terreus sensu stricto*	Italy	Toenail	No	ETest®
**10**	*A. terreus*	Italy	Toenail	No	ETest®

	sensu stricto				
**11**	*A. terreus sensu stricto*	Italy	Fingernails	No	ETest®
**12**	*A. terreus sensu stricto*	Austria	Sputum	No	ETest®
**13**	*A. terreus sensu stricto*	Austria	Sputum	Micafungin	ETest®
**14**	*A. terreus sensu stricto*	Austria	Sputum	No	ETest®
**15**	*A. terreus sensu stricto*	Austria	Sputum	No	ETest®
**16**	*A. terreus sensu stricto*	Austria	Sputum	No	ETest®
**17**	*A. terreus sensu stricto*	Austria	No data	No data	ETest®
**18**	*A. terreus sensu stricto*	Austria	BAL	No	ETest®
**19**	*A. terreus sensu stricto*	Austria	Sputum	No	ETest®
**20**	*A. terreus sensu stricto*	Austria	BAL	Voriconazole	ETest®
**21**	*Potential new species*	Italy	Biopsy	Voriconazole	ETest®
**22**	*A. hortai*	Italy	Sputum	Voriconazole	ETest®
**23**	*A. terreus sensu stricto*	Austria	Swab	No	ETest®
**24**	*A. terreus sensu stricto*	Austria	Sputum	No	ETest®
**25**	*A. terreus*	Austria	Sputum	No	ETest®

	sensu stricto				
**26**	*A. terreus sensu stricto*	Germany	Sputum	No data	ETest®
**27**	*A. citrinoterreus*	Germany	BAL	Caspofungin	ETest®
**28**	*A. terreus sensu stricto*	Germany	BAL	Caspofungin	ETest®
**29**	*A. terreus sensu stricto*	Germany	BAL	No data	ETest®
**30**	*A. terreus sensu stricto*	Germany	Sputum	No data	ETest®
**31**	*A. terreus sensu stricto*	Germany	Sputum	No data	ETest®
**32**	*A. terreus sensu stricto*	Germany	Sputum	No data	ETest®
**33**	*A. terreus sensu stricto*	Germany	Sputum	No data	ETest®
**34**	*A. terreus sensu stricto*	Germany	Sputum	No data	ETest®
**35**	*A. terreus sensu stricto*	Germany	Sputum	No data	ETest®
**36**	*A. terreus sensu stricto*	Germany	Sputum	No data	ETest®
**37**	*A. terreus sensu stricto*	Germany	Sputum	No data	ETest®
**38**	*A. terreus sensu stricto*	Germany	Sputum	No data	ETest®
**39**	*A. terreus sensu stricto*	Germany	Sputum	No data	ETest®
**40**	*A. terreus*	Germany	Sputum	No data	ETest®

	sensu stricto				
**41**	*A. terreus sensu stricto*	Germany	Sputum	No data	ETest®
**42**	*A. terreus sensu stricto*	Germany	Sputum	No data	ETest®
**43**	*A. terreus sensu stricto*	Germany	Bronchial secretion	No data	ETest®
**44**	*A. terreus sensu stricto*	Germany	Tracheal secretion	No	ETest®
**45**	*A. terreus sensu stricto*	Germany	BAL	Caspofungin	ETest®
**46**	*A. terreus sensu stricto*	Germany	BAL	No	ETest®
**47**	*A. terreus sensu stricto*	Germany	Sputum	No	ETest®
**48**	*A. terreus sensu stricto*	Germany	No data	No data	ETest®
**49**	*A. alabamensis*	Germany	Sputum	No data	ETest®
**50**	*A. terreus sensu stricto*	Austria	Swab	No data	ETest®
**51**	*A. terreus sensu stricto*	Austria	Sputum	No	ETest®
**52**	*A. terreus sensu stricto*	Austria	Sputum	No	ETest®
**53**	*A. terreus sensu stricto*	Italy	BAL	Voriconazole	ETest®
**54**	*A. citrinoterreus*	Spain	Sputum	No	ETest®

**58**	*A. terreus sensu stricto*	Austria	Sputum	No	ETest®
**59**	*A. terreus sensu stricto*	Austria	Sputum	No	ETest®
**60**	*A. terreus sensu stricto*	Austria	Sputum	No	ETest®
**61**	*A. terreus sensu stricto*	Italy	No data	No data	ETest®
**62**	*A. terreus sensu stricto*	Italy	Tracheobronchial aspirate	No data	ETest®
**63**	*A. alabamensis*	Italy	BAL	No data	ETest®
**64**	*A. terreus sensu stricto*	Italy	Sputum	Itraconazole	ETest®
**66**	*A. terreus sensu stricto*	Austria	Sputum	No	ETest®
**67**	*A. terreus sensu stricto*	Austria	Swab	No	ETest®
**69**	*A. terreus sensu stricto*	Austria	Sputum	No	ETest®
**70**	*A. terreus sensu stricto*	Austria	Swab	No	ETest®
**71**	*A. terreus sensu stricto*	Austria	Sputum	No	ETest®
**73**	*A. terreus sensu stricto*	Austria	BAL	No	ETest®
**74**	*A. terreus sensu stricto*	Austria	Nails	No	ETest®
**75**	*A. terreus sensu stricto*	Austria	Nails	No	ETest®

**77**	*A. terreus sensu stricto*	Austria	BAL	Liposomal amphotericin B, micafungin	ETest®
**78**	*A. terreus sensu stricto*	Denmark	Swab	No	ETest®
**79**	*A. terreus sensu stricto*	Denmark	BAL	No	ETest®
**80**	*A. terreus sensu stricto*	Denmark	Sputum	Itraconazole	ETest®
**81**	*A. terreus sensu stricto*	Denmark	BAL	No	ETest®
**82**	*A. terreus sensu stricto*	Denmark	BAL	No	ETest®
**83**	*A. terreus sensu stricto*	Denmark	Sputum	Posaconazole	ETest®
**84**	*A. terreus sensu stricto*	Denmark	Sputum	No	ETest®
**85**	*A. terreus sensu stricto*	Denmark	BAL	No	ETest®
**86**	*A. terreus sensu stricto*	Denmark	BAL	No	ETest®
**87**	*A. terreus sensu stricto*	Denmark	Sputum, tracheal aspirate	No	ETest®
**88**	*A. terreus sensu stricto*	Denmark	Sputum	Posaconazole	ETest®
**89**	*A. terreus sensu stricto*	Denmark	Sputum	No	ETest®
**90**	*A. terreus sensu stricto*	Denmark	BAL	No	ETest®
**91**	*A. terreus*	Denmark	Sputum	No	ETest®

	sensu stricto				
**92**	*A. terreus sensu stricto*	Denmark	Sputum	No	ETest®
**93**	*A. terreus sensu stricto*	Denmark	Aspirate	No	ETest®
**94**	*A. terreus sensu stricto*	Denmark	Sputum	Posaconazole	ETest®
**95**	*A. terreus sensu stricto*	India	BAL	No	ETest®
**96**	*A. terreus sensu stricto*	India	BAL	Itracoanzole (6 weeks along with steroids)	ETest®
**97**	*A. terreus sensu stricto*	India	Biopsy	No	ETest®
**98**	*A. terreus sensu stricto*	India	BAL	No	ETest®
**99**	*A. terreus sensu stricto*	India	Biopsy	Voriconazole	ETest®
**101**	*A. terreus sensu stricto*	India	BAL	Amphotericin B deoxycholate	ETest®
**102**	*A. terreus sensu stricto*	India	Biopsy	Itraconazole (6 weeks along with steroids)	ETest®
**103**	*A. terreus sensu stricto*	India	BAL	Itraconanzole (6 weeks along with steroids)	ETest®
**104**	*A. terreus sensu stricto*	India	Biopsy	Voriconazole	ETest®
**105**	*A. terreus sensu stricto*	India	Biopsy	Amphotericin B deoxycholate	ETest®
**106**	*A. terreus*	India	Biopsy	No	ETest®

	sensu stricto				
**107**	*A. terreus sensu stricto*	India	BAL	Itraconanzole (6 weeks along with steroids)	ETest®
**108**	*A. terreus sensu stricto*	India	Bronchial aspirate	Systemic steroids	ETest®
**110**	*A. terreus sensu stricto*	India	Sputum	No	ETest®
**111**	*A. terreus sensu stricto*	India	BAL	Systemic steroids	ETest®
**112**	*A. terreus sensu stricto*	India	Biopsy	Amphotericin B deoxycholate (two weeks followed by voriconazole next 3-4 weeks)	ETest®
**114**	*A. terreus sensu stricto*	India	BAL	Amphotericin B deoxycholate	ETest®
**115**	*A. terreus sensu stricto*	India	BAL	Voriconazole	ETest®
**116**	*A. terreus sensu stricto*	Austria	BAL	No	ETest®
**117**	*A. terreus sensu stricto*	Austria	BAL	No	ETest®
**118**	*A. terreus sensu stricto*	Texas	Biopsy	No	ETest®
**119**	*A. hortai*	Texas	Sputum	Liposomal amphotericin B	ETest®
**120**	*A. terreus sensu stricto*	Texas	No data	No data	ETest®
**121**	*A. alabamensis*	Texas	BAL	Liposomal amphotericin B	ETest®

**122**	*A. terreus sensu stricto*	Texas	BAL	Caspofungin	ETest®
**123**	*A. terreus sensu stricto*	Texas	Peritoneal fluid	Fluconazole	ETest®
**124**	*A. terreus sensu stricto*	Texas	BAL	No	ETest®
**125**	*A. terreus sensu stricto*	Texas	BAL	No	ETest®
**126**	*A. terreus sensu stricto*	Texas	Sputum	Voriconazole	ETest®
**127**	*A. terreus sensu stricto*	Texas	No data	No data	ETest®
**128**	*A. terreus sensu stricto*	India	Biopsy	Itracoanzole (6 weeks along with steroids)	ETest®
**129**	*A. terreus sensu stricto*	India	BAL	Itraconanzole (6 weeks along with steroids)	ETest®
**130**	*A. terreus sensu stricto*	Italy	Sputum	No	ETest®
**131**	*A. terreus sensu stricto*	Italy	Sputum	No	ETest®
**132**	*A. terreus sensu stricto*	Italy	No data	No data	ETest®
**133**	*A. terreus sensu stricto*	Italy	Sputum	No	ETest®
**134**	*A. terreus sensu stricto*	Italy	Sputum	No	ETest®
**135**	*A. terreus sensu stricto*	Italy	Sputum	No data	ETest®

**136**	*A. terreus sensu stricto*	Italy	Sputum	No	ETest®
**137**	*A. terreus sensu stricto*	Austria	No data	No data	ETest®
**138**	*A. terreus sensu stricto*	Spain	Sputum	Posaconazole	ETest®
**139**	*A. terreus sensu stricto*	Spain	Sputum	Posaconazole	ETest®
**140**	*A. citrinoterreus*	Spain	Sputum	No	ETest®
**141**	*A. terreus sensu stricto*	Spain	Sputum	Voriconazole	ETest®
**142**	*A. hortai*	Spain	BAL	Liposomal amphotericin B	ETest®
**143**	*A. terreus sensu stricto*	Spain	Sputum	Voriconazole	ETest®
**144**	*A. terreus sensu stricto*	Spain	BAL	Nebulized amphotericin B (profilaxis), voriconazole	ETest®
**145**	*A. terreus sensu stricto*	Spain	Sputum	No	ETest®
**146**	*A. terreus sensu stricto*	Spain	Sputum	No	ETest®
**147**	*A. terreus sensu stricto*	Spain	Sputum	No	ETest®
**148**	*A. citrinoterreus*	Spain	Sputum	Voriconazole	ETest®
**149**	*A. citrinoterreus*	Spain	Sputum	Voriconazole	ETest®

**150**	*A. citrinoterreus*	Spain	Sputum	Voriconazole	ETest®
**151**	*A. terreus sensu stricto*	Spain	Sputum	No	ETest®
**152**	*A. terreus sensu stricto*	Spain	Bronchial aspirate	Nebulized amphotericin B, micafungin	ETest®
**153**	*A. terreus sensu stricto*	Spain	Sputum	No	ETest®
**154**	*A. terreus sensu stricto*	Spain	Sputum	No	ETest®
**155**	*A. terreus sensu stricto*	Spain	Sputum	No	ETest®
**156**	*A. terreus sensu stricto*	Spain	Sputum	Nebulized amphotericin B, posaconazole	ETest®
**157**	*A. terreus sensu stricto*	Spain	Sputum	Voriconazole	ETest®
**158**	*A. hortai*	Spain	Sputum	Voriconazole	ETest®
**159**	*A. terreus sensu stricto*	Spain	Sputum	No	ETest®
**160**	*A. terreus sensu stricto*	Spain	Sputum	No	ETest®
**161**	*A. terreus sensu stricto*	Spain	Sputum	No	ETest®
**162**	*A. alabamensis*	Spain	Sputum	Liposomal amphotericin B	ETest®
**163**	*A. terreus sensu stricto*	Spain	Bronchial aspirate	Liposomal amphotericin B. voriconazole	ETest®

**164**	*A. terreus sensu stricto*	Spain	Tracheal aspirate	Nebulized amphotericin B (profilax), voriconazole; liposomal amphotericin B, voriconazole	ETest®
**165**	*A. citrinoterreus*	Spain	Sputum	Posaconazole	ETest®
**166**	*A. citrinoterreus*	Spain	Sputum	Posaconazole	ETest®
**167**	*A. terreus sensu stricto*	Spain	Sputum	No	ETest®
**168**	*A. terreus sensu stricto*	Spain	Sputum	Nebulized amphotericin B, posaconazole	ETest®
**169**	*A. terreus sensu stricto*	Spain	Sputum	Posaconazole	ETest®
**170**	*A. terreus sensu stricto*	Spain	Sputum	Posaconazole	ETest®
**171**	*A. terreus sensu stricto*	Sweden	Sputum	No	ETest®
**172**	*A. terreus sensu stricto*	Sweden	BAL	No	ETest®
**173**	*A. terreus sensu stricto*	Spain	Sputum	Voriconazole	ETest®
**174**	*A. terreus sensu stricto*	Spain	Sputum	Nebulized amhotericin B	ETest®
**175**	*A. citrinoterreus*	Spain	BAL	Micafungin	ETest®
**176**	*A. citrinoterreus*	Spain	Sputum	No	ETest®

**177**	*A. terreus sensu stricto*	Spain	Sputum	No	ETest®
**178**	*A. terreus sensu stricto*	Spain	Sputum	No	ETest®
**179**	*A. terreus sensu stricto*	Spain	Sputum	No	ETest®
**180**	*A. terreus sensu stricto*	Spain	Bronchial aspirate	No	ETest®
**181**	*A. alabamensis*	Spain	Sputum	No	ETest®
**182**	*A. citrinoterreus*	Spain	Sputum	No	ETest®
**183**	*A. terreus sensu stricto*	Spain	Sputum	No	ETest®
**184**	*A. terreus sensu stricto*	Spain	Bronchial aspirate	No	ETest®
**185**	*A. terreus sensu stricto*	Spain	Sputum	No	ETest®
**186**	*A. terreus sensu stricto*	Spain	Sputum	No	ETest®
**188**	*A. terreus sensu stricto*	Spain	Sputum	No	ETest®
**189**	*A. citrinoterreus*	Spain	Bronchial aspirate	No	ETest®
**190**	*A. terreus sensu stricto*	Spain	Bronchial aspirate	No	ETest®
**191**	*A. terreus sensu stricto*	Spain	Sputum	No	ETest®
**192**	*A. terreus sensu stricto*	Belgium	Sputum	No	ETest®

**193**	*A. terreus sensu stricto*	Belgium	Sputum	No	ETest®
**194**	*A. terreus sensu stricto*	Belgium	BAL	No	ETest®
**196**	*A. terreus sensu stricto*	Spain	Sputum	No	ETest®
**197**	*A. terreus sensu stricto*	Serbia	Sputum	No	ETest®
**198**	*A. terreus sensu stricto*	Serbia	Sputum	No	ETest®
**199**	*A. terreus sensu stricto*	Turkey	Swab	No	ETest®
**200**	*A. terreus sensu stricto*	Turkey	Swab	No	ETest®
**201**	*A. terreus sensu stricto*	Turkey	BAL	No	ETest®
**202**	*A. terreus sensu stricto*	Turkey	Tracheal aspirate	Caspofungin	ETest®
**203**	*A. terreus sensu stricto*	Turkey	Swab	Liposomal amphotericin B	ETest®
**204**	*A. floccosus*	Turkey	Swab	Liposomal amphotericin B	ETest®
**205**	*A. citrinoterreus*	Turkey	Tracheal aspirate	No	ETest®
**206**	*A. terreus sensu stricto*	Turkey	Swab	No	ETest®
**207**	*A. terreus sensu stricto*	Germany	Sputum	No	ETest®
**208**	*A. terreus sensu stricto*	Austria	No data	No data	ETest®

**209**	*A. terreus sensu stricto*	Austria	No data	No	ETest®
**210**	*A. terreus sensu stricto*	Netherlands	BAL	No	ETest®
**211**	*A. terreus sensu stricto*	Netherlands	BAL	No	ETest®
**212**	*A. terreus sensu stricto*	Netherlands	Swab	No	ETest®
**213**	*A. terreus sensu stricto*	Netherlands	Sputum	No	ETest®
**214**	*A. terreus sensu stricto*	Netherlands	Sputum	No	ETest®
**215**	*A. terreus sensu stricto*	Netherlands	Sputum	No	ETest®
**216**	*A. terreus sensu stricto*	Netherlands	Sputum	No	ETest®
**217**	*A. citrinoterreus*	Netherlands	Sputum	No	ETest®
**218**	*A. terreus sensu stricto*	Netherlands	Sputum	No	ETest®
**219**	*A. terreus sensu stricto*	Greece	BAL	No	ETest®
**220**	*A. citrinoterreus*	Greece	Nails	No	ETest®
**221**	*A. terreus sensu stricto*	Greece	Swab	No	ETest®
**222**	*A. terreus sensu stricto*	no data	No data	No data	ETest®
**223**	*A. terreus sensu stricto*	United Kingdom	Blood	No	ETest®

**224**	*A. terreus sensu stricto*	United Kingdom	BAL	Voriconazole	ETest®
**225**	*A. alabamensis*	United Kingdom	Swab	No	ETest®
**226**	*A. terreus sensu stricto*	United Kingdom	Swab	No	ETest®
**227**	*A. terreus sensu stricto*	United Kingdom	Sputum	No	ETest®
**228**	*A. citrinoterreus*	United Kingdom	Sputum	No	ETest®
**229**	*A. citrinoterreus*	United Kingdom	BAL	Voriconazole	ETest®
**230**	*A. terreus sensu stricto*	Qatar	Sputum	No	ETest®
**231**	*A. terreus sensu stricto*	Qatar	Swab	No	ETest®
**232**	*A. terreus sensu stricto*	Qatar	Sputum	No	ETest®
**233**	*A. citrinoterreus*	Qatar	Swab	No	ETest®
**234**	*A. citrinoterreus*	Qatar	Endotracheal aspirate	No	ETest®
**235**	*A. terreus sensu stricto*	Qatar	BAL	No	ETest®
**236**	*A. terreus sensu stricto*	Qatar	Sputum	No	ETest®
**237**	*A. terreus sensu stricto*	Qatar	Swab	Clotrimazole 1%	ETest®
**238**	*A. terreus sensu stricto*	Qatar	BAL	Anidulafungin	ETest®

**239**	*A. terreus sensu stricto*	Qatar	Swab	No	ETest®
**240**	*A. terreus sensu stricto*	Qatar	Swab	No	ETest®
**241**	*A. terreus sensu stricto*	Qatar	Sputum	Anidulafungin	ETest®
**242**	*A. terreus sensu stricto*	Qatar	Swab	No	ETest®
**243**	*A. citrinoterreus*	Qatar	Biopsy	Itraconazole	ETest®
**244**	*A. terreus sensu stricto*	Qatar	Sputum	No	ETest®
**245**	*A. terreus sensu stricto*	Qatar	Swab	Clotrimazole 1%	ETest®
**246**	*A. terreus sensu stricto*	Qatar	BAL	No	ETest®
**247**	*A. terreus sensu stricto*	Qatar	Bronchial wash	No	ETest®
**248**	*A. terreus sensu stricto*	Germany	No data	No data	ETest®
**249**	*A. terreus sensu stricto*	Spain	Sputum	No	ETest®
**250**	*A. terreus sensu stricto*	Spain	Sputum	No	ETest®
**251**	*A. terreus sensu stricto*	Spain	Nails	No	ETest®
**252**	*A. terreus sensu stricto*	Spain	Sputum	Voriconazole	ETest®
**253**	*A. terreus sensu stricto*	Spain	Sputum	No	ETest®

**254**	*A. terreus sensu stricto*	Spain	Nails	No	ETest®
**255**	*A. terreus sensu stricto*	Spain	Sputum	No	ETest®
**256**	*A. citrinoterreus*	Spain	Sputum	Voriconazole	ETest®
**257**	*A. terreus sensu stricto*	Spain	Sputum	No	ETest®
**258**	*A. terreus sensu stricto*	Spain	Sputum	Voriconazole	ETest®
**259**	*A. terreus sensu stricto*	Spain	Sputum	No	ETest®
**260**	*A. citrinoterreus*	Spain	Sputum	No	ETest®
**261**	*A. terreus sensu stricto*	Spain	Sputum	No	ETest®
**262**	*A. neoafricanus*	Spain	Nails	No	ETest®
**263**	*A. citrinoterreus*	Spain	Nails	No	ETest®
**264**	*A. terreus sensu stricto*	Spain	Sputum	Voriconazole	ETest®
**265**	*A. terreus sensu stricto*	Spain	Sputum	Itraconazole	ETest®
**266**	*A. terreus sensu stricto*	Spain	Sputum	No	ETest®
**267**	*A. terreus sensu stricto*	Spain	Sputum	No	ETest®
**268**	*A. terreus sensu stricto*	Spain	Nails	No	ETest®

**269**	*A. terreus sensu stricto*	Spain	Nails	No	ETest®
**270**	*A. terreus sensu stricto*	Spain	Nails	No	ETest®
**271**	*A. terreus sensu stricto*	Spain	Nails	No	ETest®
**272**	*A. terreus sensu stricto*	Spain	Nails	No	ETest®
**273**	*A. terreus sensu stricto*	Spain	Sputum	Voriconazole	ETest®
**274**	*A. terreus sensu stricto*	Spain	Nails	No	ETest®
**275**	*A. citrinoterreus*	Spain	Sputum	No	ETest®
**276**	*A. terreus sensu stricto*	Spain	Nails	No	ETest®
**277**	*A. citrinoterreus*	Spain	Nails	No	ETest®
**278**	*A. citrinoterreus*	Iran	Biopsy	Amphotericin B	ETest®
**279**	*A. citrinoterreus*	Iran	BAL	No data	ETest®
**280**	*A. citrinoterreus*	Iran	Biopsy	No data	ETest®
**281**	*A. citrinoterreus*	Iran	Sinuses discharge	No data	ETest®
**282**	*A. terreus sensu stricto*	Iran	Environment (swab)	No data	ETest®
**283**	*A. terreus sensu stricto*	Iran	Swab	Itraconazole	ETest®

**284**	*A. terreus sensu stricto*	Iran	BAL	Itraconazole	ETest®
**285**	*A. terreus sensu stricto*	Iran	Biopsy	No data	ETest®
**286**	*A. terreus sensu stricto*	Iran	Swab	Itraconazole	ETest®
**287**	*A. terreus sensu stricto*	Iran	Biopsy	No data	ETest®
**288**	*A. terreus sensu stricto*	Iran	Swab	Itraconazole	ETest®
**289**	*A. terreus sensu stricto*	Austria	No data	No data	ETest®
**290**	*A. citrinoterreus*	Austria	No data	No data	ETest®
**291**	*A. terreus sensu stricto*	Austria	No data	No data	ETest®
**292**	*A. terreus sensu stricto*	Austria	No data	No data	ETest®
**293**	*A. terreus sensu stricto*	Austria	No data	No data	ETest®
**294**	*A. terreus sensu stricto*	Austria	No data	No data	ETest®
**295**	*A. terreus sensu stricto*	Austria	No data	No data	ETest®
**296**	*A. terreus sensu stricto*	Austria	No data	No data	ETest®
**297**	*A. citrinoterreus*	Czech Republic	Swab	No	ETest®
**298**	*A. terreus sensu stricto*	Czech Republic	BAL	Itraconazole	ETest®

**299**	*A. terreus sensu stricto*	Czech Republic	Swab	No	ETest®
**300**	*A. hortai*	Czech Republic	Swab	No	ETest®
**301**	*A. terreus sensu stricto*	Czech Republic	Swab	No	ETest®
**302**	*A. hortai*	Czech Republic	Sputum	No	ETest®
**303**	*A. terreus sensu stricto*	Czech Republic	Tracheal secretion	Voriconazole	ETest®
**304**	*A. terreus sensu stricto*	Czech Republic	No data	No data	ETest®
**305**	*A. terreus sensu stricto*	Czech Republic	No data	No data	ETest®
**306**	*A. hortai*	Czech Republic	No data	No data	ETest®
**307**	*A. terreus sensu stricto*	Czech Republic	No data	No data	ETest®
**308**	*A. citrinoterreus*	Israel	No data	No data	ETest®
**309**	*A. citrinoterreus*	Israel	No data	No data	ETest®
**310**	*A. terreus sensu stricto*	Israel	No data	No data	ETest®
**311**	*A. terreus sensu stricto*	Israel	No data	No data	ETest®
**312**	*A. terreus sensu stricto*	Israel	No data	No data	ETest®
**313**	*A. terreus sensu stricto*	Israel	No data	No data	ETest®

**315**	*A. terreus sensu stricto*	Israel	No data	No data	ETest®
**316**	*A. terreus sensu stricto*	Israel	No data	No data	ETest®
**317**	*A. terreus sensu stricto*	Israel	No data	No data	ETest®
**318**	*A. terreus sensu stricto*	Israel	No data	No data	ETest®
**319**	*A. terreus sensu stricto*	Israel	No data	No data	ETest®
**320**	*A. hortai*	Israel	No data	No data	ETest®
**321**	*A. terreus sensu stricto*	Israel	No data	No data	ETest®
**322**	*A. terreus sensu stricto*	Israel	No data	No data	ETest®
**323**	*A. terreus sensu stricto*	Israel	No data	No data	ETest®
**324**	*A. citrinoterreus*	Israel	No data	No data	ETest®
**325**	*A. terreus sensu stricto*	Israel	No data	No data	ETest®
**326**	*A. terreus sensu stricto*	Israel	No data	No data	ETest®
**327**	*A. terreus sensu stricto*	Israel	No data	No data	ETest®
**328**	*A. terreus sensu stricto*	Israel	No data	No data	ETest®
**329**	*A. terreus sensu stricto*	Israel	No data	No data	ETest®

**330**	*A. terreus sensu stricto*	Israel	No data	No data	ETest®
**331**	*A. terreus sensu stricto*	Israel	No data	No data	ETest®
**332**	*A. citrinoterreus*	Israel	No data	No data	ETest®
**333**	*A. terreus sensu stricto*	Israel	No data	No data	ETest®
**334**	*A. terreus sensu stricto*	Israel	No data	No data	ETest®
**335**	*A. terreus sensu stricto*	Israel	No data	No data	ETest®
**336**	*A. terreus sensu stricto*	Israel	No data	No data	ETest®
**337**	*A. terreus sensu stricto*	Israel	No data	No data	ETest®
**338**	*A. terreus sensu stricto*	Israel	No data	No data	ETest®
**339**	*A. terreus sensu stricto*	Israel	No data	No data	ETest®
**340**	*A. terreus sensu stricto*	Israel	No data	No data	ETest®
**341**	*A. terreus sensu stricto*	Israel	No data	No data	ETest®
**342**	*A. terreus sensu stricto*	No data	No data	No data	ETest®
**343**	*A. terreus sensu stricto*	Austria	No data	No data	ETest®
**344**	*A. terreus sensu stricto*	Austria	No data	No data	ETest®

**345**	*A. terreus sensu stricto*	Austria	No data	No data	ETest®
**346**	*A. terreus sensu stricto*	Austria	No data	No data	ETest®
**347**	*A. terreus sensu stricto*	Austria	No data	No data	ETest®
**348**	*A. terreus sensu stricto*	Austria	No data	No data	ETest®
**349**	*A. hortai*	Italy	No data	No data	ETest®
**350**	*A. terreus sensu stricto*	Italy	Toenail		ETest®
**351**	*A. hortai*	Brazil	No data	No data	ETest®
**352**	*A. terreus sensu stricto*	Brazil	Environment (swab)	No data	ETest®
**353**	*A. terreus sensu stricto*	Brazil	No data	No data	ETest®
**354**	*A. terreus sensu stricto*	Brazil	No data	No data	ETest®
**355**	*A. terreus sensu stricto*	Brazil	No data	No data	ETest®
**356**	*A. terreus sensu stricto*	Brazil	No data	No data	ETest®
**357**	*A. terreus sensu stricto*	Brazil	No data	No data	ETest®
**358**	*A. terreus sensu stricto*	France	Sputum	No data	ETest®
**359**	*A. terreus sensu stricto*	France	Sputum	No data	ETest®

**360**	*A. terreus sensu stricto*	France	Sputum	No data	ETest®
**361**	*A. terreus sensu stricto*	France	Sputum	No data	ETest®
**362**	*A. terreus sensu stricto*	France	Sputum	No data	ETest®
**363**	*A. terreus sensu stricto*	France	Sputum	No data	ETest®
**364**	*A. terreus sensu stricto*	France	Sputum	No data	ETest®
**365**	*A. terreus sensu stricto*	France	Sputum	No data	ETest®
**366**	*A. terreus sensu stricto*	France	Sputum	No data	ETest®
**367**	*A. terreus sensu stricto*	France	Sputum	No data	ETest®
**368**	*A. terreus sensu stricto*	France	Sputum	No data	ETest®
**369**	*A. terreus sensu stricto*	Austria	No data	No data	ETest®
**371**	*A. terreus sensu stricto*	Austria	No data	No data	ETest®
**372**	*A. terreus sensu stricto*	Austria	No data	No data	ETest®
**373**	*A. terreus sensu stricto*	Greece	Sputum	Liposomales amphotericin B	ETest®
**374**	*A. terreus sensu stricto*	Greece	Sputum	Liposomales amphotericin B	ETest®
**375**	*A. terreus sensu stricto*	Greece	Sputum	Liposomales amphotericin B	ETest®

**376**	*A. terreus sensu stricto*	Portugal	BAL	No data	ETest®
**377**	*A. terreus sensu stricto*	Portugal	BAL	No data	ETest®
**378**	*A. terreus sensu stricto*	Czech Republic	Puncture	No	ETest®
**379**	*A. terreus sensu stricto*	Czech Republic	Swab	No	ETest®
**380**	*A. terreus sensu stricto*	Spain	Nails	No	ETest®
**381**	*A. terreus sensu stricto*	Spain	Sputum	Voriconazole	ETest®
**382**	*A. terreus sensu stricto*	Spain	Surgical injury	No	ETest®
**383**	*A. terreus sensu stricto*	Spain	Sputum	No	ETest®
**384**	*A. terreus sensu stricto*	Czech Republic	No data	No data	ETest®
**T10**	*A. terreus sensu stricto*	Austria	No data	No data	EUCAST
**T100**	*A. terreus sensu stricto*	Austria	Sputum	No data	EUCAST
**T101**	*A. terreus sensu stricto*	Austria	BAL	No data	EUCAST
**T102**	*A. terreus sensu stricto*	Austria	BAL	No data	EUCAST
**T103**	*A. terreus sensu stricto*	Austria	Sputum	No data	EUCAST
**T104**	*A. terreus sensu stricto*	UK	No data	No data	EUCAST

**T105**	*A. citrinoterreus*	Austria	Sputum	No data	EUCAST
**T106**	*A. terreus sensu stricto*	Austria	Sputum	No data	EUCAST
**T107**	*A. terreus sensu stricto*	Austria	BAL	No data	EUCAST
**T109**	*A. alabamensis*	Netherlands	No data	No data	EUCAST
**T11**	*A. terreus sensu stricto*	Austria	No data	No data	EUCAST
**T110**	*A. alabamensis*	Netherlands	No data	No data	EUCAST
**T112**	*A. terreus sensu stricto*	Germany	Swab	No data	EUCAST
**T113**	*A. terreus sensu stricto*	Austria	No data	No data	EUCAST
**T12**	*A. terreus sensu stricto*	Austria	No data	No data	EUCAST
**T128**	*A. terreus sensu stricto*	Germany	Bronhial secretion	No data	EUCAST
**T129**	*A. citrinoterreus*	Germany	Swab	No data	EUCAST
**T13**	*A. terreus sensu stricto*	Austria	No data	No data	EUCAST
**T130**	*A. terreus sensu stricto*	Austria	BAL	No data	EUCAST
**T131**	*A. terreus sensu stricto*	Austria	Sputum	No data	EUCAST
**T132**	*A. terreus sensu stricto*	Austria	No data	No data	EUCAST

**T134**	*A. terreus sensu stricto*	Austria	No data	No data	EUCAST
**T135**	*A. terreus sensu stricto*	Austria	No data	No data	EUCAST
**T136**	*A. terreus sensu stricto*	Germany	Nails	No data	EUCAST
**T139**	*A. hortai*	Germany	Swab	No data	EUCAST
**T14**	*A. terreus sensu stricto*	Austria	No data	No data	EUCAST
**T140**	*A. terreus sensu stricto*	Germany	Swab	No data	EUCAST
**T141**	*A. terreus sensu stricto*	Austria	No data	No data	EUCAST
**T144**	*A. terreus sensu stricto*	Germany	Bronhial secretion	No data	EUCAST
**T146**	*A. terreus sensu stricto*	Austria	No data	No data	EUCAST
**T147**	*A. terreus sensu stricto*	Germany	Swab	No data	EUCAST
**T148**	*A. terreus sensu stricto*	Germany	Swab	No data	EUCAST
**T149**	*A. citrinoterreus*	Germany	Swab	No data	EUCAST
**T15**	*A. terreus sensu stricto*	Austria	No data	No data	EUCAST
**T150**	*A. citrinoterreus*	Germany	No data	No data	EUCAST
**T152**	*A. terreus sensu stricto*	Austria	No data	No data	EUCAST

**T153**	*A. terreus sensu stricto*	Austria	No data	No data	EUCAST
**T154**	*A. terreus sensu stricto*	Austria	No data	No data	EUCAST
**T155**	*A. terreus sensu stricto*	Austria	No data	No data	EUCAST
**T156**	*A. terreus sensu stricto*	Germany	No data	No data	EUCAST
**T157**	*A. terreus sensu stricto*	Austria	No data	No data	EUCAST
**T158**	*A. terreus sensu stricto*	Austria	No data	No data	EUCAST
**T159**	*A. terreus sensu stricto*	Austria	No data	No data	EUCAST
**T16**	*A. terreus sensu stricto*	Austria	No data	No data	EUCAST
**T160**	*A. terreus sensu stricto*	Germany	No data	No data	EUCAST
**T17**	*A. terreus sensu stricto*	Austria	No data	No data	EUCAST
**T18**	*A. terreus sensu stricto*	Austria	No data	No data	EUCAST
**T19**	*A. terreus sensu stricto*	Austria	No data	No data	EUCAST
**T2**	*A. terreus sensu stricto*	Austria	No data	No data	EUCAST
**T20**	*A. terreus sensu stricto*	Austria	No data	No data	EUCAST
**T21**	*A. terreus sensu stricto*	Austria	No data	No data	EUCAST

**T22**	*A. terreus sensu stricto*	Austria	Sputum	No data	EUCAST
**T24**	*A. terreus sensu stricto*	Austria	No data	No data	EUCAST
**T25**	*A. terreus sensu stricto*	Austria	No data	No data	EUCAST
**T26**	*A. terreus sensu stricto*	Austria	No data	No data	EUCAST
**T27**	*A. terreus sensu stricto*	Austria	No data	No data	EUCAST
**T28**	*A. terreus sensu stricto*	Austria	No data	No data	EUCAST
**T29**	*A. terreus sensu stricto*	Austria	No data	No data	EUCAST
**T3**	*A. terreus sensu stricto*	Austria	No data	No data	EUCAST
**T30**	*A. terreus sensu stricto*	Austria	No data	No data	EUCAST
**T31**	*A. terreus sensu stricto*	Austria	No data	No data	EUCAST
**T32**	*A. terreus sensu stricto*	Austria	BAL	No data	EUCAST
**T34**	*A. terreus sensu stricto*	Austria	BAL	No data	EUCAST
**T35**	*A. terreus sensu stricto*	Austria	Sputum	No data	EUCAST
**T36**	*A. terreus sensu stricto*	Austria	Biopsy	No data	EUCAST
**T4**	*A. terreus sensu stricto*	Austria	No data	No data	EUCAST

**T40**	*A. terreus sensu stricto*	Austria	No data	No data	EUCAST
**T41**	*A. terreus sensu stricto*	Austria	No data	No data	EUCAST
**T42**	*A. terreus sensu stricto*	Austria	No data	No data	EUCAST
**T43**	*A. terreus sensu stricto*	Austria	Biopsy	No data	EUCAST
**T44**	*A. terreus sensu stricto*	Austria	Bronchial secretion	No data	EUCAST
**T47**	*A. terreus sensu stricto*	Austria	No data	No data	EUCAST
**T49**	*A. terreus sensu stricto*	Austria	Environment (swab)	No data	EUCAST
**T5**	*A. terreus sensu stricto*	Austria	No data	No data	EUCAST
**T50**	*A. terreus sensu stricto*	Austria	Swab	No data	EUCAST
**T51**	*A. terreus sensu stricto*	Austria	BAL	No data	EUCAST
**T52**	*A. terreus sensu stricto*	Germany	No data	No data	EUCAST
**T53**	*A. hortai*	Germany	No data	No data	EUCAST
**T54**	*A. terreus sensu stricto*	Germany	No data	No data	EUCAST
**T55**	*A. terreus sensu stricto*	Germany	No data	No data	EUCAST
**T56**	*A. terreus sensu stricto*	Germany	No data	No data	EUCAST

**T57**	*A. terreus sensu stricto*	Germany	No data	No data	EUCAST
**T58**	*A. terreus sensu stricto*	Germany	No data	No data	EUCAST
**T59**	*A. terreus sensu stricto*	Germany	No data	No data	EUCAST
**T6**	*A. terreus sensu stricto*	Austria	No data	No data	EUCAST
**T60**	*A. terreus sensu stricto*	Germany	No data	No data	EUCAST
**T61**	*A. terreus sensu stricto*	Austria	BAL	No data	EUCAST
**T62**	*A. terreus sensu stricto*	Austria	Bronchial secretion	No data	EUCAST
**T63**	*A. terreus sensu stricto*	Austria	Biopsy	No data	EUCAST
**T64**	*A. terreus sensu stricto*	Austria	Swab	No data	EUCAST
**T65**	*A. terreus sensu stricto*	Austria	Sputum	No data	EUCAST
**T66**	*A. hortai*	Austria	Swab	No data	EUCAST
**T67**	*A. terreus sensu stricto*	Austria	Bronchial secretion	No data	EUCAST
**T68**	*A. terreus sensu stricto*	Austria	BAL	No data	EUCAST
**T69**	*A. terreus sensu stricto*	Austria	BAL	No data	EUCAST
**T7**	*A. terreus sensu stricto*	Austria	No data	No data	EUCAST

**T70**	*A. citrinoterreus*	Austria	No data	No data	EUCAST
**T71**	*A. terreus sensu stricto*	Austria	BAL	No data	EUCAST
**T72**	*A. terreus sensu stricto*	Austria	BAL	No data	EUCAST
**T73**	*A. terreus sensu stricto*	Austria	Biopsy	No data	EUCAST
**T74**	*A. terreus sensu stricto*	Spain	No data	No data	EUCAST
**T75**	*A. terreus sensu stricto*	Spain	No data	No data	EUCAST
**T76**	*A. terreus sensu stricto*	Germany	Swab	No data	EUCAST
**T77**	*A. terreus sensu stricto*	Italy	Swab	No data	EUCAST
**T78**	*A. terreus sensu stricto*	Italy	No data	No data	EUCAST
**T79**	*A. terreus sensu stricto*	Italy	No data	No data	EUCAST
**T8**	*A. terreus sensu stricto*	Austria	No data	No data	EUCAST
**T80**	*A. terreus sensu stricto*	Italy	No data	No data	EUCAST
**T81**	*A. terreus sensu stricto*	Italy	No data	No data	EUCAST
**T82**	*A. terreus sensu stricto*	Italy	No data	No data	EUCAST
**T83**	*A. terreus sensu stricto*	Italy	No data	No data	EUCAST

**T84**	*A. terreus sensu stricto*	Italy	No data	No data	EUCAST
**T85**	*A. terreus sensu stricto*	Italy	No data	No data	EUCAST
**T86**	*A. terreus sensu stricto*	Italy	No data	No data	EUCAST
**T87**	*A. terreus sensu stricto*	Italy	No data	No data	EUCAST
**T88**	*A. terreus sensu stricto*	Italy	No data	No data	EUCAST
**T89**	*A. terreus sensu stricto*	Italy	No data	No data	EUCAST
**T9**	*A. terreus sensu stricto*	Austria	No data	No data	EUCAST
**T90**	*A. citrinoterreus*	Italy	No data	No data	EUCAST
**T91**	*A. terreus sensu stricto*	Germany	Swab	No data	EUCAST
**T92**	*A. terreus sensu stricto*	Germany	Swab	No data	EUCAST
**T93**	*A. terreus sensu stricto*	Austria	Bronchial secretion	No data	EUCAST
**T94**	*A. terreus sensu stricto*	Austria	Biopsy	No data	EUCAST
**T95**	*A. terreus sensu stricto*	Austria	Sputum	No data	EUCAST
**T96**	*A. terreus sensu stricto*	Austria	Biopsy	No data	EUCAST
**T97**	*A. terreus sensu stricto*	Austria	BAL	No data	EUCAST

**T98**	*A. terreus sensu stricto*	Austria	BAL	No data	EUCAST
**T99**	*A. terreus sensu stricto*	Austria	No data	No data	EUCAST

EUCAST. European Committee for Antimicrobial Susceptibility. BAL. bronchoalveolar lavage.

Supplementary Table S2. Wild type reference strains used for *cyp51a* sequencing for *Aspergillus terreus* and *Aspergillus citrinoterreus*

**Wild type strain *Aspergillus terreus***	NIH2624
**Wild type strain *Aspergillus citrinoterreus***	3 (environmental azole susceptible isolate)

Supplementary Table S3. Primers for *Cyp51A* sequencing used in study

**Primer**	**Sequence**	**Purpose**
**cyp51-AT-0**	5’-GGTGGGAGAACTTTCGTTCTA-3’	Amplification and sequencing
**cyp51-AT-3**	5’-CTCCATACAAACCACTGACCTC-3’	Sequencing
**cyp51-AT-4**	5’-ACGGCAGCATGAAGTTGAT-3’	Sequencing
**cyp51-AT-5**	5’-CGAGTTTGCCGACCTCTAC-3’	Sequencing
**cyp51-AT-8**	5’-GCCGTTCTTACGCCTTGT-3’	Amplification and sequencing

Supplementary Figure S1. Results of molecular identification (partial beta-tubulin sequencing)

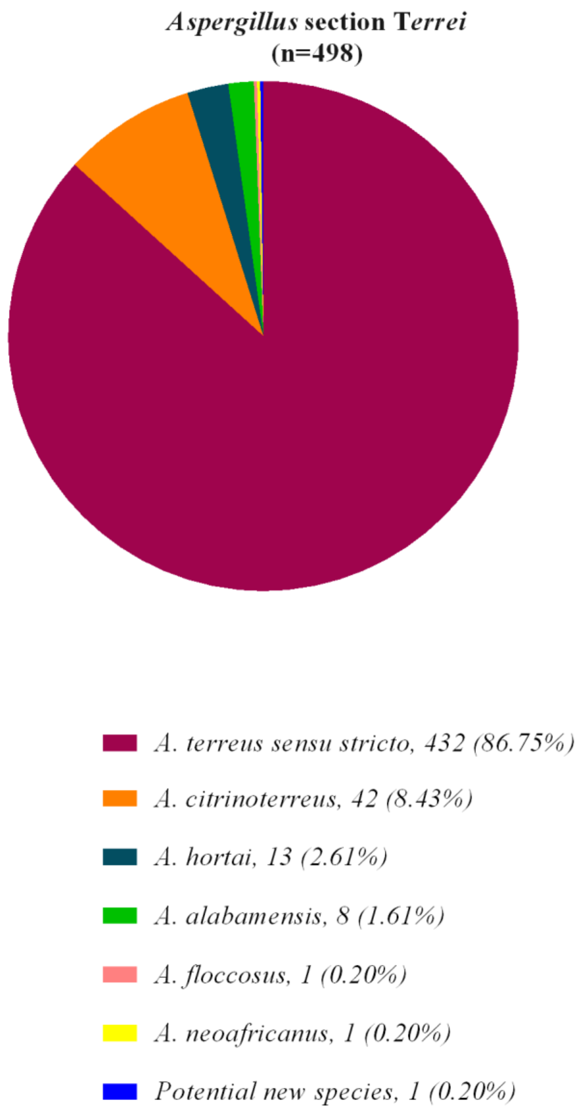

Supplementary Figure S2 (a-d). Overview of geographical origin of section *Terrei* isolates per species: a) *A. terreus sensu stricto*, b) *A. citrinoterreus*, c) *A. hortai*, and d) *A. alabamensis*

a)

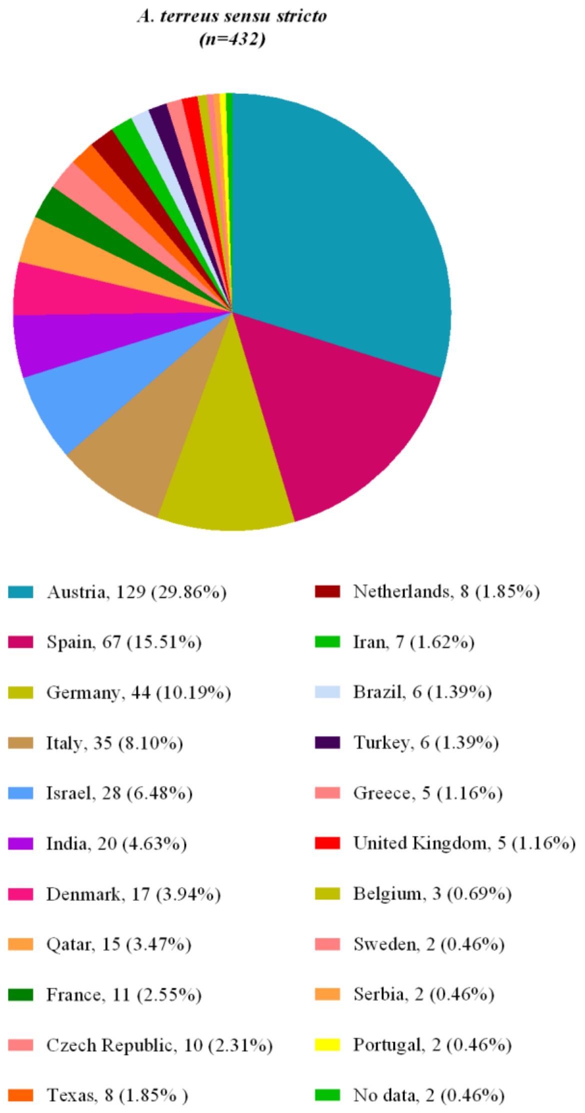

b)

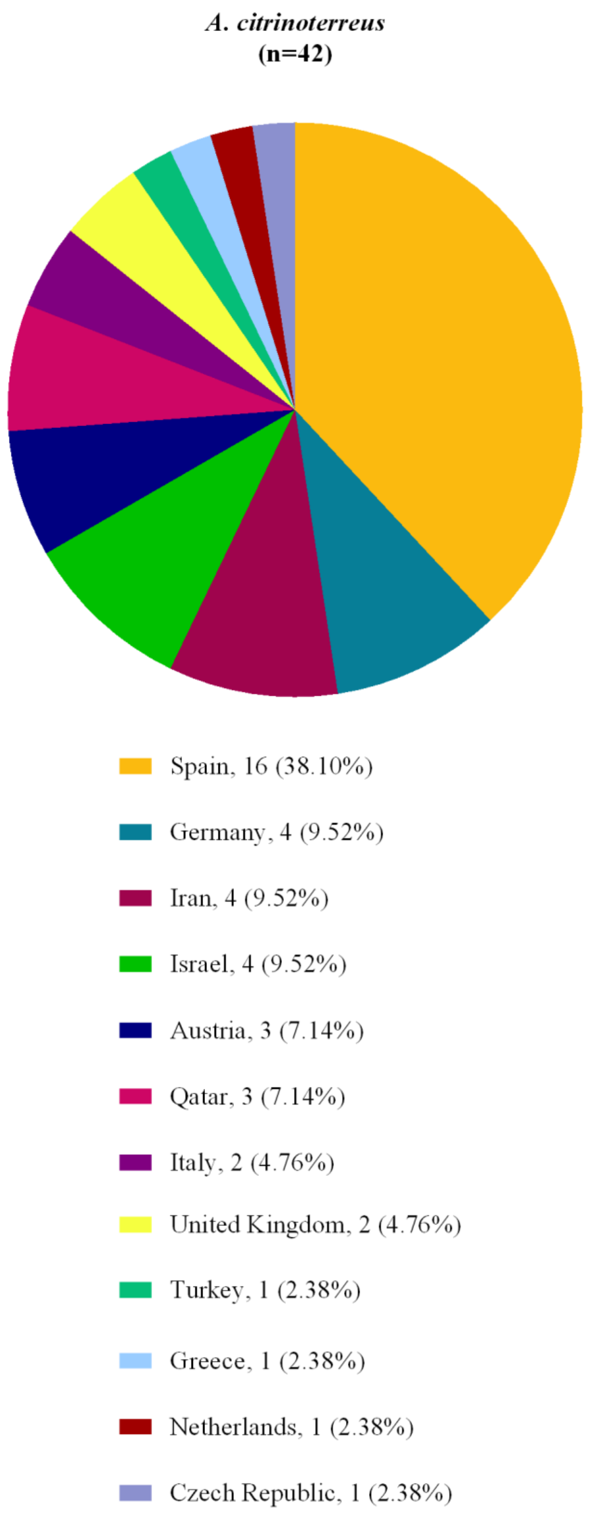
A. citrinoterreus
(n=42)
Spain, 16 (38.10%)
Germany, 4 (9.52%)
Iran, 4 (9.52%)
Israel, 4 (9.52%)
Austria, 3 (7.14%)
Qatar, 3 (7.14%)
Italy, 2 (4.76%)
United Kingdom, 2 (4.76%)
Turkey, 1 (2.38%)
Greece, 1 (2.38%)
Netherlands, 1 (2.38%)
Czech Republic, 1 (2.38%)

c)

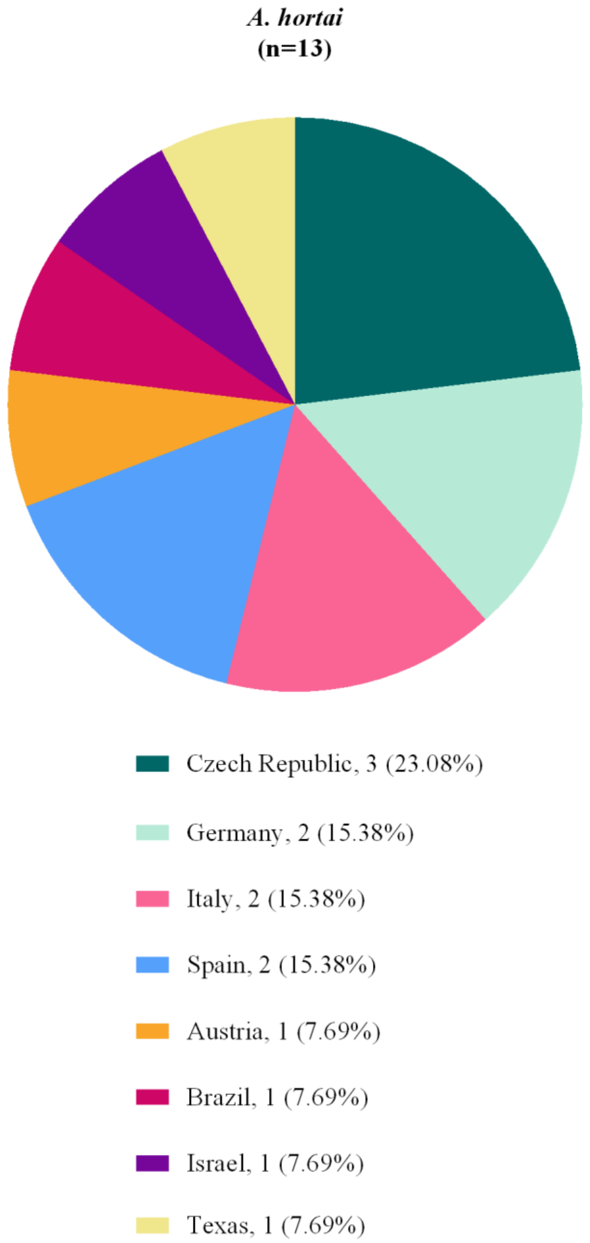
A. hortai
(n=13)
Czech Republic, 3 (23.08%)
Germany, 2 (15.38%)
Italy, 2 (15.38%)
Spain, 2 (15.38%)
Austria, 1 (7.69%)
Brazil, 1 (7.69%)
Israel, 1 (7.69%)
Texas, 1 (7.69%)

d)

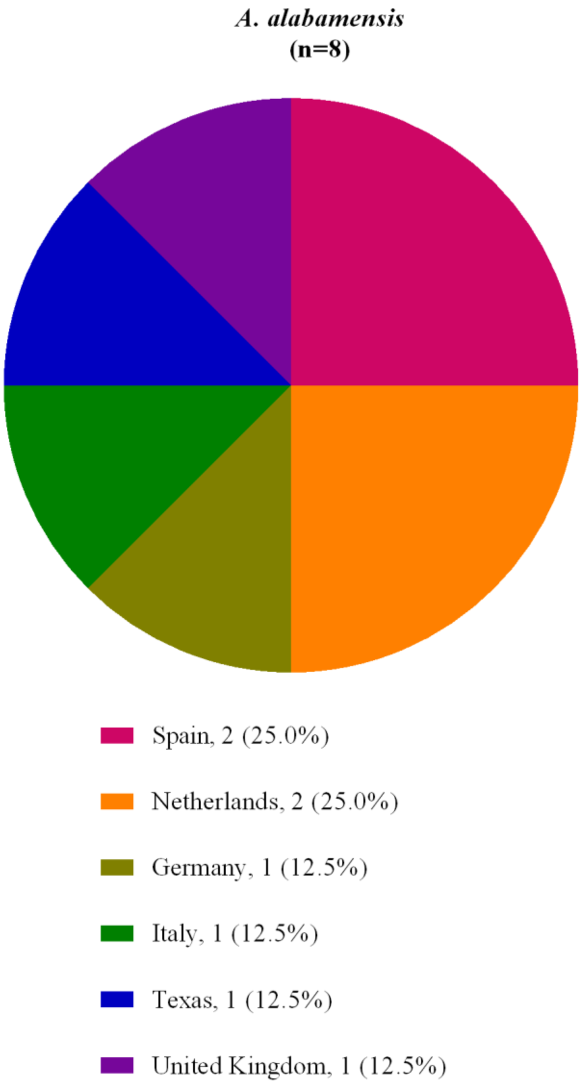
A. alabamensis
(n=8)
Spain, 2 (25.0%)
Netherlands, 2 (25.0%)
Germany, 1 (12.5%)
Italy, 1 (12.5%)
Texas, 1 (12.5%)
United Kingdom, 1 (12.5%)
